# Reproductive history determines *Erbb2* locus amplification, WNT signalling and tumour phenotype in a murine breast cancer model

**DOI:** 10.1242/dmm.048736

**Published:** 2021-05-18

**Authors:** Liliana D. Ordonez, Lorenzo Melchor, Kirsty R. Greenow, Howard Kendrick, Giusy Tornillo, James Bradford, Peter Giles, Matthew J. Smalley

**Affiliations:** 1 European Cancer Stem Cell Research Institute and Cardiff School of Biosciences, Cardiff University, Hadyn Ellis Building, Maindy Road, Cardiff CF24 4HQ, UK; 2 Almac Diagnostic Services, Craigavon BT63 5QD, UK; 3 Wales Gene Park, University Hospital Wales, Heath Park, Cardiff CF14 4XW, UK

**Keywords:** HER2, *NeuNT*, *Erbb2*, WNT, Pregnancy, Breast cancer

## Abstract

Understanding the mechanisms underlying tumour heterogeneity is key to the development of treatments that can target specific tumour subtypes. We have previously targeted CRE recombinase-dependent conditional deletion of the tumour suppressor genes *Brca1*, *Brca2*, *p53* (also known as *Trp53*) and/or *Pten* to basal or luminal oestrogen receptor-negative (ER^−^) cells of the mouse mammary epithelium. We demonstrated that both the cell-of-origin and the tumour-initiating genetic lesions cooperate to influence mammary tumour phenotype. Here, we use a CRE-activated HER2 orthologue to specifically target HER2/ERBB2 oncogenic activity to basal or luminal ER^−^ mammary epithelial cells and perform a detailed analysis of the tumours that develop. We find that, in contrast to our previous studies, basal epithelial cells are less sensitive to transformation by the activated *NeuKI* allele, with mammary epithelial tumour formation largely confined to luminal ER^−^ cells. Histologically, most tumours that developed were classified as either adenocarcinomas of no special type or as metaplastic adenosquamous tumours. The former were typically characterized by amplification of the *NeuNT/Erbb2* locus; in contrast, tumours displaying squamous metaplasia were enriched in animals that had been through at least one pregnancy and typically had lower levels of *NeuNT/Erbb2* locus amplification but had activated canonical WNT signalling. Squamous changes in these tumours were associated with activation of the epidermal differentiation cluster. Thus, in this model of HER2 breast cancer, cell-of-origin, reproductive history, *NeuNT/Erbb2* locus amplification and the activation of specific branches of the WNT signalling pathway all interact to drive inter-tumour heterogeneity.

## Abstract (precis)

**Summary:** Using a mouse model of breast cancer, the authors show mammary epithelial cell-type sensitivity to transformation by HER2, as well as a change in tumour phenotype associated with reproductive history and driven by WNT signalling.

## INTRODUCTION

The ERBB2/HER2 receptor tyrosine kinase (a member of the epidermal growth factor receptor family) is amplified and overexpressed in 20-30% of human breast cancers, leading to an aggressive form of the disease (Andrulis et al., 1998). Therapeutic strategies that block HER2 activity, either with antibodies targeted to the extracellular domain or with small molecules blocking intracellular kinase activity, have substantial benefit (Nielsen et al., 2013), although primary or acquired resistance remains an issue (Garrett and Arteaga, 2011). HER2 activity has been associated with stem cell-like behaviour in normal breast and breast cancer (Ithimakin et al., 2013; Korkaya et al., 2008), but the cell-of-origin of HER2 breast cancers remains unclear.

Understanding how cell-of-origin and genetic lesions interact to drive tumour behaviour and heterogeneity is key to the development of treatments that can be targeted to specific tumour subtypes. In the mammary epithelium, potential cells of tumour origin include basal cells, luminal oestrogen receptor-negative (ER^−^) progenitors and luminal ER^+^ cells (largely differentiated hormone-sensing cells, although including a small proportion of progenitors) (Soady et al., 2015). In previous studies of mammary tumour origin, we used genetically engineered mouse models in which CRE recombinase-dependent conditional deletion of the tumour suppressor genes *Brca1*, *Brca2*, *p53* (also known as *Trp53*) and/or *Pten* were targeted to basal or luminal ER^−^ cells using the *Krt14* or *Blg* promoters, respectively. We demonstrated that both the cell-of-origin and the tumour-initiating genetic lesions cooperate to influence the tumour behaviour, with a more diverse range of tumour phenotypes arising from luminal ER^−^ cells but basal-origin tumours having significantly shorter latency (Melchor et al., 2014; Molyneux et al., 2010).

Here, we have addressed whether cell-of-origin similarly affects the development and phenotype of *HER2*-amplified tumours. We have taken advantage of the *NeuKI* allele, in which an activated mutant variant of *Neu* (*NeuNT*), the rat *Erbb2/Her2* orthologue, has been knocked into the endogenous *Erbb2* locus. *NeuNT* is therefore expressed under the control of the endogenous promoter but only when an upstream *loxP-*flanked neomycin cassette is excised by CRE recombinase activity (Andrechek et al., 2000). Using this allele, we have been able to target NeuNT activity to basal or luminal ER^−^ cells using our established *Krt14Cre* and *BlgCre* lines. We have performed a detailed analysis of *Krt14Cre-NeuKI* and *BlgCre-NeuKI* mice, assessing tumours from virgin and parous animals. For comparison, we have used both normal non-transgenic tissue and tumours from the *MMTV-NeuNDL* model, in which *Neu* expression is driven constitutively by a very strong mammary promoter (Siegel et al., 1999).

We find that, in contrast to our previous studies, basal epithelial cells are less sensitive to transformation by the activated *NeuKI* allele, with mammary epithelial tumour formation enriched in luminal ER^−^ cells. Most tumours arising from the latter population were classified as either adenocarcinomas of no special type [AC(NST)] or metaplastic adenosquamous carcinomas (ASQC). Remarkably, the proportion of ASQCs arising from this cell type depended on the reproductive history of the animal, with ASQC tumours strongly associated with animals that had been through at least one pregnancy. Furthermore, whereas AC(NST) tumours were typically characterized by high amplification of the *NeuNT/Erbb2* locus, ASQC tumours typically had lower levels of *NeuNT/Erbb2* locus amplification but activated canonical WNT signalling and the set of genes known as the epidermal differentiation cluster (EDC).

Thus, reproductive history affects *NeuNT/Erbb2* locus amplification and the activation of specific branches of the WNT signalling pathway and ultimately drives inter-tumour heterogeneity in this murine model of human HER2 breast cancer. Importantly, our findings also demonstrate that different cell types can be differentially sensitive to transformation by particular oncogenic drivers, suggesting at least one mechanism why certain mutations only cause cancer in a distinct range of target organs.

## RESULTS

### *Erbb2*/*Neu* is expressed in preneoplastic *Krt14Cre*-*NeuKI* and *BlgCre*-*NeuKI* mammary epithelium at comparable levels to wild type

To assess baseline levels of endogenous *Erbb2* expression in pre-neoplastic tissue from the mouse lines in this study, we stained sections of wild-type, *Krt14Cre-NeuKI* and *BlgCre-NeuKI* mammary fat pads from 10-week-old virgin mice (Fig. S1B). There were no obvious differences in staining of the mammary epithelium between these samples; however, immunohistochemistry is difficult to accurately quantify. Therefore, we used our standard flow cytometric protocols (Soady et al., 2015) to isolate mammary epithelial cell subpopulations (basal stem cells, myoepithelial cells, luminal ER^−^ progenitors and luminal ER^+^ differentiated cells) from 10- to 12-week-old (before any evidence of tumour formation) *MMTV-NeuNDL*, *Krt14Cre-NeuKI* and *BlgCre-NeuKI*, as well as wild-type C57Bl6, mice. Comparison of relative expression levels of endogenous *Erbb2* in each mammary epithelial subpopulation showed that endogenous *Erbb2* was expressed at lower levels in the transgenic/knock-in lines than in the wild-type cells in three of the four populations, the exception being the luminal ER^+^ cells, in which it was higher in the transgenic/knock-in lines than in the wild-type cells (Fig. S1C). These differences may result from a combination of the design/expression of the genetic constructs in the transgenic/knock-in lines, as well as the difference in background strains (the wild-type cells were from C57/BL6, whereas the genetically modified mice were a mixed background).

### Luminal progenitors are more sensitive and basal cells less sensitive to *NeuKI* allele-dependent tumourigenesis

*Krt14Cre* targets CRE expression/activity primarily to basal cells and *BlgCre* primarily to luminal ER^−^ cells in the mammary epithelium (Molyneux et al., 2010). To test the contribution of cell-of-origin to tumour phenotype in HER2 mouse models, cohorts of *Krt14Cre-NeuKI*, *BlgCre-NeuKI* and *MMTV-NeuNDL* mice were aged, together with a control group carrying the *NeuKI* allele but no *Cre* transgene. A subset of the *BlgCre* and *Krt14Cre* cohorts underwent one or more pregnancies.

**Fig. 1.**
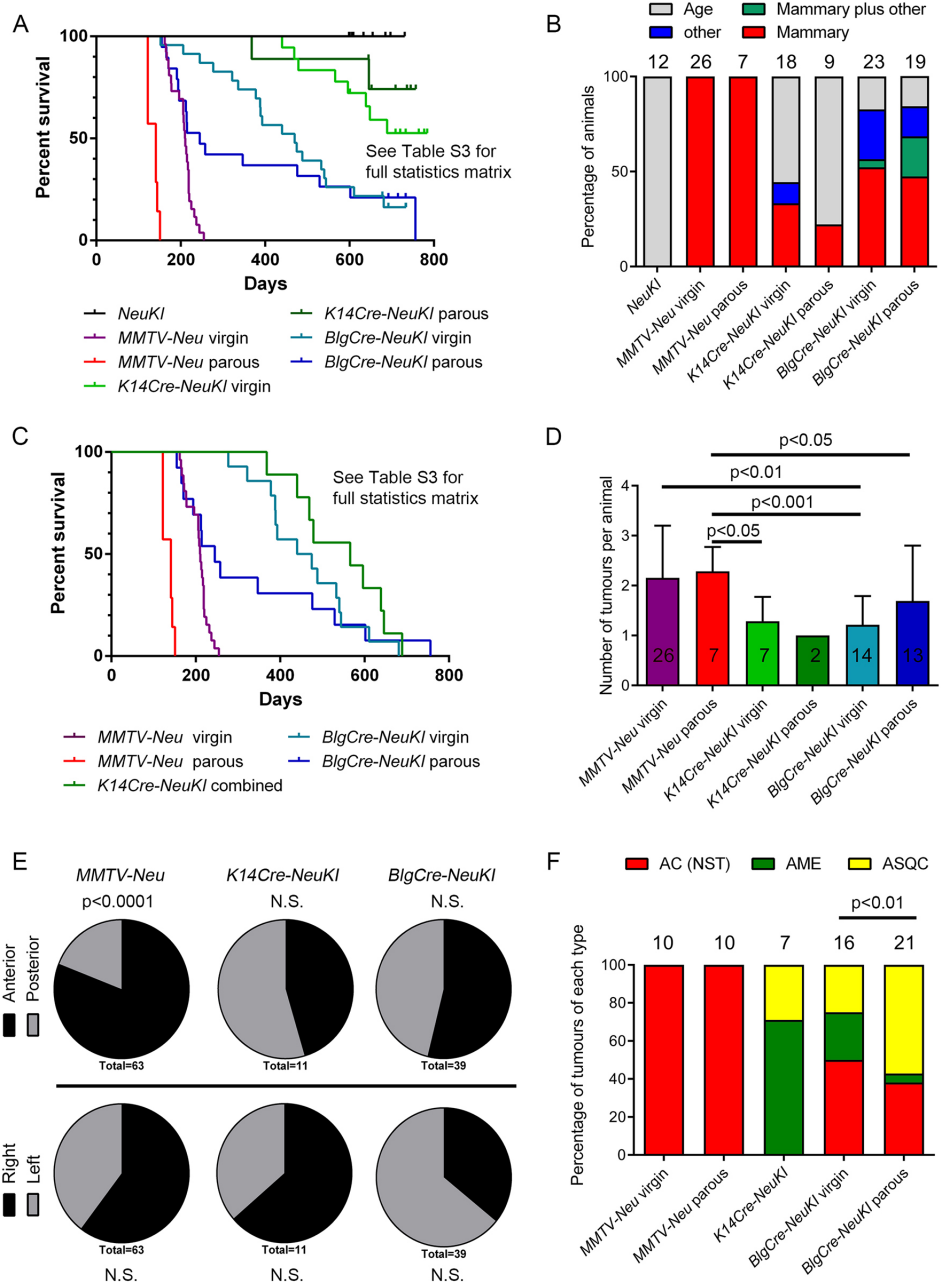
**Features of tumour cohorts.** (A) Kaplan–Meier survival curves (time to euthanasia for any reason) for all cohorts. Statistical significances between the cohorts is provided in full in Table S3. (B) Reason for euthanasia as a proportion of cohort. ‘Age’: no phenotype/mice had reached 2 years of age. ‘Other’: tumours developed elsewhere than the mammary gland (head/neck). Numbers of mice in each cohort are indicated. (C) Kaplan–Meier curves of mammary tumour latency. Full statistics are provided in Table S3. Owing to small numbers, data for virgin and parous *K14Cre-NeuKI* cohorts are combined. (D) Numbers of tumours per animal (data are mean±s.d.). The total number of animals assessed is indicated. Full statistical information is provided in Table S3. (E) Body location of mammary epithelial tumours. The number of tumours assessed is indicated (which may include more than one tumour from one animal). Anterior, tumours developing in the 1st/2nd/3rd mammary fat pads; posterior, tumours developing in the 4th/5th fat pads. Right/left, tumours developing on the right/left-hand side when viewing the animal from the dorsal aspect. (F) Distribution of mammary epithelial tumour histopathological phenotypes. The number of tumours in each group undergoing detailed histological analysis is indicated. AC(NST), adenocarcinoma (no special type); AME, adenomyoepithelioma; ASQC, adenosquamous carcinoma. Sarcomas arising in the mammary gland and elsewhere are not included. Data for virgin and parous *K14Cre-NeuKI* tumours are combined. Full details of all tumours are provided in Table S4. N.S., not significant.

Full details of the animals in the cohorts are provided in Table S1. These included 33 *MMTV-NeuNDL* mice (26 virgin, seven parous), 42 *BlgCre-NeuKI* mice (23 virgin, 19 parous), 27 *K14Cre-NeuKI* mice (18 virgin, nine parous) and 12 control mice carrying the *NeuKI* allele but no *Cre* transgene (all virgin). Kaplan–Meier curves for overall survival are shown in Fig. 1A and statistical comparisons are provided in Table S3. The penetrance of the different phenotypes within each line is shown in Fig. 1B.

Control cohort animals survived more than 600 days, whereas both parous and virgin *MMTV-NeuNDL* mice rapidly succumbed to mammary tumours (median latencies 141 and 210 days, respectively). *BlgCre-NeuKI* mice survived longer than *MMTV-NeuNDL* mice (*P*<0.0001, Log Rank Mantel–Cox; *P*<0.0001, Gehan–Breslow–Wilcoxon) but had a significantly shorter survival than *K14Cre-NeuKI* mice (*P*=0.0017, Log Rank Mantel–Cox; *P*=0.0007, Gehan–Breslow–Wilcoxon comparing the virgin mice of each cohort) (*P*=0.0024, Log Rank Mantel–Cox; *P*=0.0030, Gehan–Breslow–Wilcoxon comparing the parous mice of each cohort) (Fig. 1A; Table S3). Indeed, the majority of *K14Cre-NeuKI* mice were culled due to age rather than pathology, whereas *BlgCre-NeuKI* mice developed tumours in a number of sites, mainly the mammary gland but also on the head/neck (some *BlgCre-NeuKI* mice were also culled due to age; Fig. 1B). This is in strong contrast to our previous studies on mice carrying conditional *Brca1*, *p53* or *Pten* alleles, in which mice carrying the *K14Cre* transgene had a significantly shorter survival than *BlgCre* mice (Melchor et al., 2014; Molyneux et al., 2010).

Parity significantly accelerated tumour onset in *MMTV-Neu* mice (*P*<0.0001, Log Rank Mantel–Cox; *P*<0.0001, Gehan–Breslow–Wilcoxon). It also lowered median overall survival and tumour-specific survival in *BlgCre* mice (median overall survival: virgin animals, 470 days; parous animals, 245 days; tumour-specific survival: virgin animals, 457 days; parous animals, 245 days); however, the overall survival effects were not significant, and the significance of the difference in age of mammary tumour onset depended on the statistical test used (*BlgCre* virgin versus parous overall survival, *P*=0.4109 not significant, Log Rank Mantel–Cox; *P*=0.0809 not significant, Gehan–Breslow–Wilcoxon) (*BlgCre* virgin versus parous tumour-specific survival, *P*=0.2619 not significant, Log Rank Mantel–Cox; *P*=0.0204, Gehan–Breslow–Wilcoxon) (Fig. 1C; Table S3). The median tumour-specific survival of the combined *K14Cre-NeuKI* cohort was 566 days.

Therefore, whereas our previous findings established that basal mammary cells were more sensitive than luminal cells to loss of *Brca1/2*, *Pten* and *p53* (Melchor et al., 2014; Molyneux et al., 2010), here we demonstrate that luminal cells are more sensitive to activation of the *NeuKI* allele and that the basal cell population is less sensitive to the tumour-promoting activity of this allele.

### Parity alters tumour phenotype in the *BlgCre*-*NeuKI* model

We next focused on the numbers and locations of mammary tumours in the tumour cohorts on a mouse-by-mouse basis, as well as on the tumour phenotypes. Given the small number of mammary tumours from the *Krt14Cre-NeuKI* model, data from the virgin and parous cohorts of this line were combined.

Mice from the *MMTV-NeuNDL* cohorts developed, on average, 2.2 tumours per animal, whereas *Krt14Cre-NeuKI* mice and *BlgCre-NeuKI* virgin animals developed 1.3 and 1.2 tumours per animal, respectively (Fig. 1D; Table S3). There was no significant difference in mean tumour number per animal between *BlgCre-NeuKI* parous mice (1.7) and either the virgin mice of the same cohort or the *MMTV-NeuNDL* mice. However, there was a wide range in the numbers of tumours per animal seen in the *BlgCre-NeuKI* parous cohort (Table S1), suggesting that, although pregnancy was having a biological effect on this cohort, it was not consistent between animals.

Breast cancer shows a laterality bias, with ∼10% higher incidence in the left breast (Amer, 2014; Ekbom et al., 1994; Senie et al., 1980). We therefore analysed mouse necropsy data for evidence of a locational bias in tumour origins. For this analysis, virgin and parous cohorts were combined and we categorized tumours as originating on either the left or right side of the mouse or in the anterior or posterior mammary glands (mammary glands 1, 2 and 3 being anterior, 4 and 5 being posterior) (Fig. 1E). There was no significant left-right bias in tumour origins in any of the mouse lines. There was no significant anterior-posterior bias in the *Krt14Cre-NeuKI* and *BlgCre-NeuKI* lines. However, in the *MMTV-NeuNDL* line, tumours were significantly (*P*<0.0001) more likely to develop from the anterior, as opposed to the posterior, mammary glands.

**Fig. 2.**
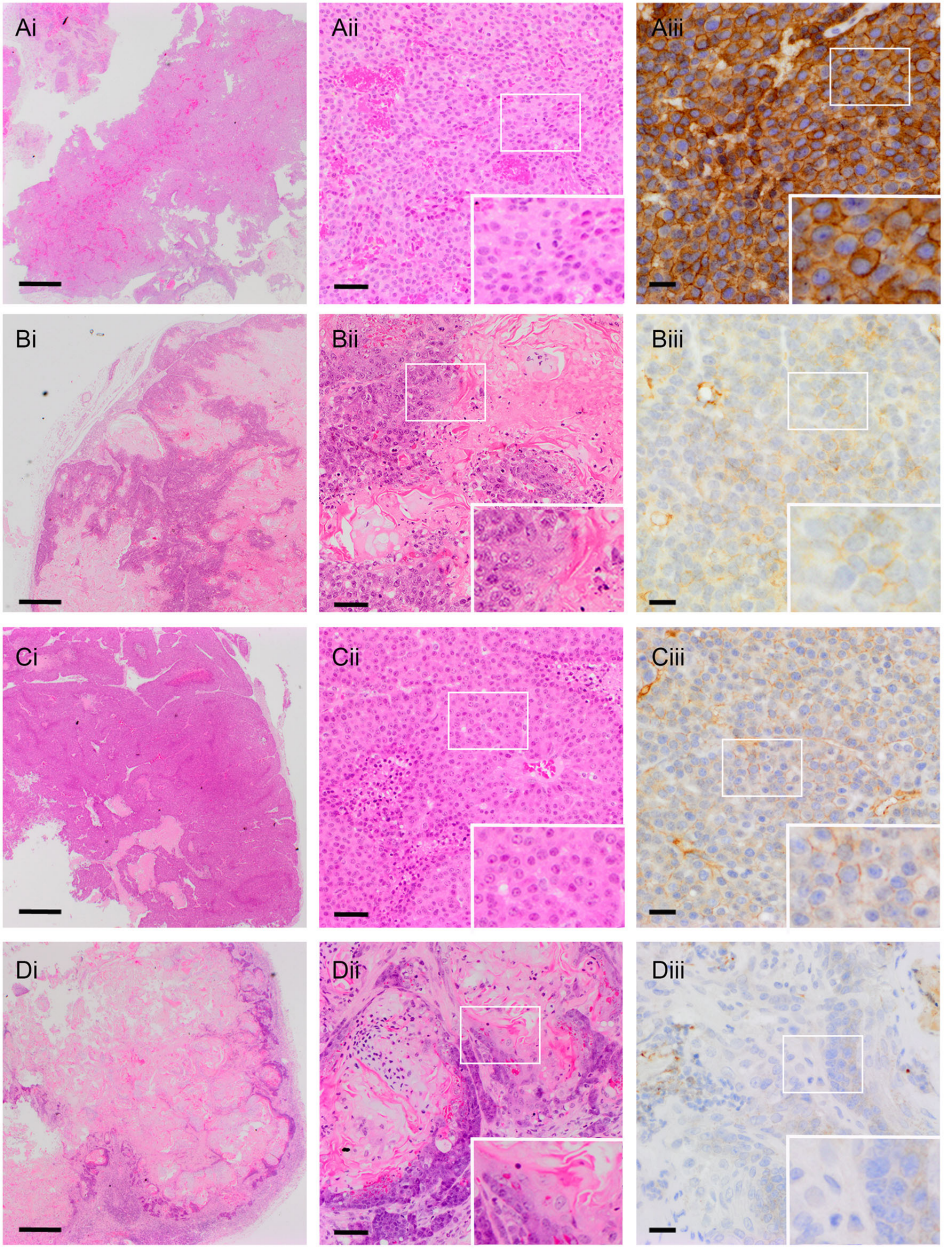
**Tumour histology.** (A-Diii) Representative low-magnification (i) and high-magnification (ii) H&E images, and ERBB2 staining (iii) of (A) a virgin *MMTV-NeuNDL* AC(NST) tumour, (B) a virgin *Krt14Cre-NeuKI* ASQC tumour, (C) a virgin *BlgCre-NeuKI* AC(NST) tumour and (D) a parous *BlgCre-NeuKI* ASQC tumour. Insets are at twice the magnification of the boxed region in each panel. Scale bars: 500 µm in i; 50 µm in ii; 20 µm in iii.

For the analysis of tumour phenotypes, we undertook a detailed comparative study as previously described (Melchor et al., 2014; Molyneux et al., 2010). In brief, mouse mammary tumours fall into four main histotypes: adenomyoepithelioma (AME), metaplastic adenosquamous carcinoma (ASQC), metaplastic spindle cell carcinoma (MSCC) and adenocarcinoma of no special type [AC(NST)], all of which can be readily diagnosed from Haematoxylin and Eosin (H&E) and ΔNp63 staining (which identifies key differential diagnostic features, i.e. the presence of metaplastic features and the number/pattern of ΔNp63-stained cells). Full details of the analysis are given in Table S4. Ten virgin and ten parous *MMTV-Neu* tumours were analysed. These were invariably diagnosed as AC(NST) (Fig. 1F). They grew as sheets of epithelioid cells; only one showed evidence of metaplasia. In these tumours, ERBB2 showed, as expected, extremely strong membrane staining (Fig. 2A).

Six virgin and one parous *K14Cre-NeuKI* tumours were available for histological analysis. The low numbers resulted from the cystic nature of many of these tumours, which collapsed upon excision and left little or no material for embedding. Of those that could be analysed, five were diagnosed as AMEs and two as ASQC tumours (Fig. 1F), consistent with our previous findings on phenotypes of tumours arising from the basal populations. Four of these tumours were available for ERBB2 staining. One parous tumour showed no ERBB2 staining; in one virgin tumour, staining was punctate within the cytoplasm; in two virgin tumours, weak membrane staining could be seen (Fig. 2B).

Analysis of the *BlgCre-NeuKI* tumours was the most striking. Seventeen virgin and 23 parous tumours arising in the mammary gland were available for analysis. One of the virgin and two of the parous tumours were diagnosed as malignant mammary sarcomas and excluded from the analysis (the head/neck tumours were also diagnosed as sarcomas). Of the remainder, 50% (*n*=8) virgin tumours were classed as AC(NST), 25% (*n*=4) as AMEs and 25% (*n*=4) as ASQCs. In contrast, 38% (*n*=8) of parous tumours were AC(NST), 5% (*n*=1) were AME and 57% (*n*=12) were ASQCs. This was a statistically significant increase in ASQC tumours in parous compared with virgin *BlgCre-NeuKI* mice (Fig. 1F). The change in proportion of AC(NST) tumours compared with non-AC(NST) tumours in virgin versus parous mice was not significant (*P*=0.077, χ^2^ test), indicating that the shift to the ASQC phenotype was largely at the expense of the AME phenotype. In contrast to the strong membrane staining seen in *MMTV-Neu* tumours, ERBB2 staining in *BlgCre-NeuKI* tumours was weak and typically cytoplasmic, with weak membrane staining in fewer than half of all cases (Fig. 2C,D). In some tumours, ERBB2 staining was undetectable. No MSCCs were observed in any cohort.

Importantly, the parous and virgin *BlgCre-NeuKI* cohorts were established from littermates randomly assigned to the groups and therefore of identical background genetics. Thus, in this model, developmental history of the mammary gland has a biological effect on tumour phenotype, with parity favouring the formation of ASQC tumours.

### AC(NST) tumours show the largest copy number gain at the *Erbb2*/*Neu* locus

**Fig. 3.**
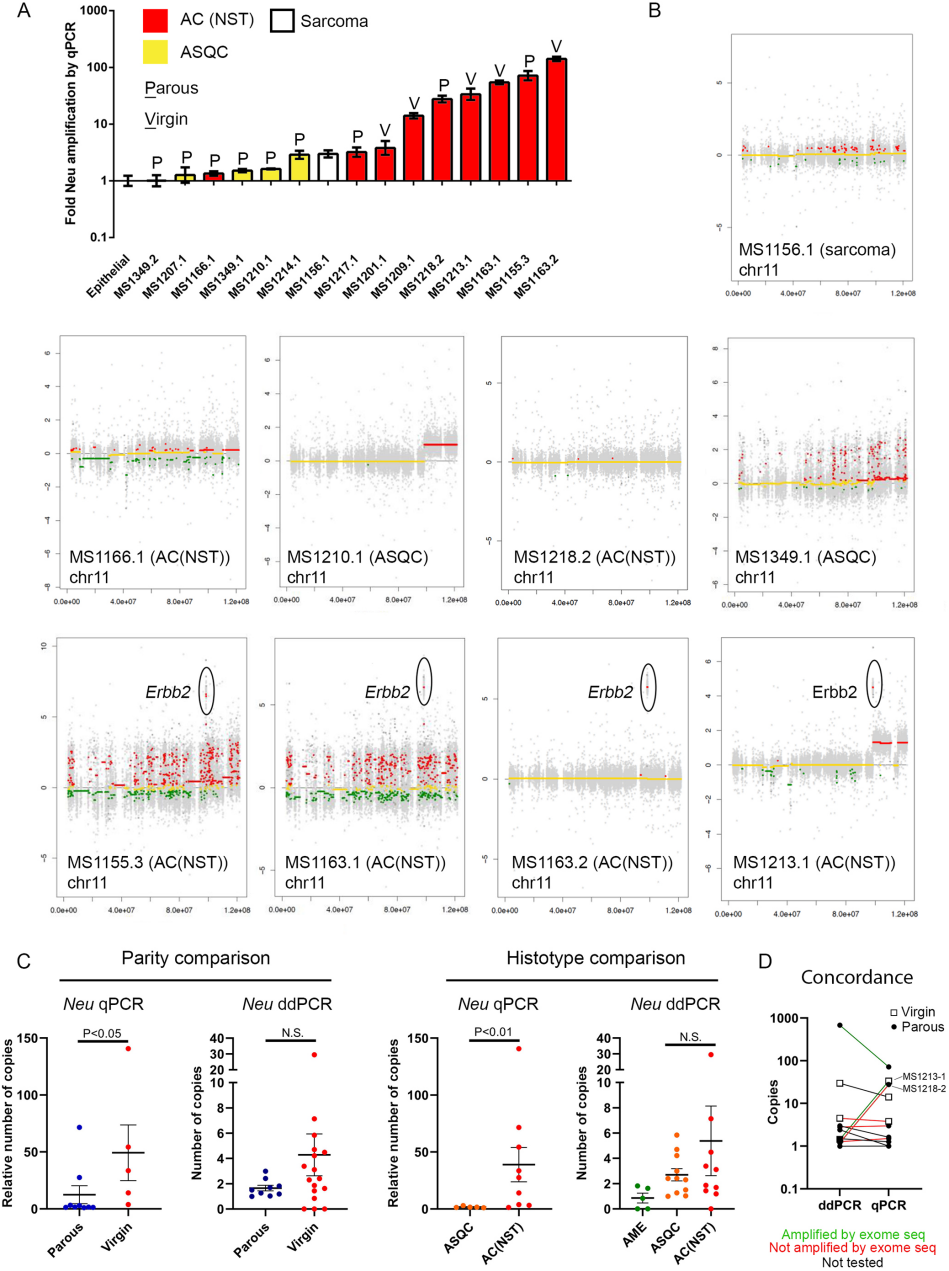
***Neu* allele amplification in *BlgCre-NeuKI* tumours.** (A) *Neu* allele amplification assessed by qPCR on tumour genomic DNA relative to control normal primary mammary epithelial cells. Data are mean±95% confidence intervals of three technical replicates on each tumour. Parity status and tumour histotype are indicated. (B) CNV-by-exome analysis of chromosome 11 in nine tumours from the *BlgCre-NeuKI* cohort. Tumour type and amplification of the *Erbb2* locus are indicated. See Table S7 for the full results in detail and Table S8 for a summary of the significant changes identified. (C) Comparison of *Neu* amplification status (data are mean±s.e.m.; two-tailed *t*-test with Welch's correction on Log10-transformed values) by parity and histotype as assessed by qPCR and ddPCR. Parity status/histotypes are indicated. (D) Concordance in findings between tumours analysed for *Neu* copy number by both ddPCR and qPCR, with CNV-by-exome findings indicated by the colour of the connecting line where available. The majority of tumours have similar copy number levels when assessed by both methods, although there is more variability in tumours with the very highest values. However, two tumours score ‘low’ Neu by ddPCR but ‘high’ Neu by qPCR. One of these was found to be amplified, while the other was not amplified, by CNV-by-exome, arguing against a systematic error in the methods and in favour of sampling errors relating to tumour heterogeneity. N.S., not significant.

Tumour formation in a mouse model in which the *NeuKI* allele was activated by an *MMTV-Cre* transgene was associated with amplification of this locus (Andrechek et al., 2000). To test whether the *Neu* allele was also amplified in tumours from the *BlgCre*-*NeuKI* model, we tested a subset of tumours with a quantitative PCR (qPCR) approach using a *Neu*-specific probe on genomic tumour DNA. The tumours consisted of a mixture of AC(NSTs) and ASQCs (nine versus five) that had come from both virgin and parous animals (five versus nine). Additionally, one non-epithelial tumour (a mammary sarcoma) was included (Fig. 3A). We found that the *Erbb2/Neu* locus was typically most highly amplified in AC(NSTs) relative to control DNA from normal primary mammary cells. In contrast, in ASQC tumours and the sarcoma, there were lower levels of amplification, although some AC(NST)s (e.g. MS1166.1) also fell within the low amplification group.

We selected ten tumours that were copy number profiled by qPCR, together with matched spleens, for exome sequencing. The tumour samples used for molecular analysis are summarized in Table S5, detailed exome sequencing results are provided in Table S6 and a summary of the results in Table S7. DNA/RNA isolated from one ASQC sample (MS1207.1) was of insufficient quality for sequencing. The remaining nine tumours consisted of six AC(NST), two ASQC tumours and a sarcoma. The nine tumours had a median of 278 mutations, both coding and non-coding (range 167-636). There was a median of 19 coding mutations (range 13-33) predicted to alter protein expression (Table S7). However, only 14 genes were mutated in more than one sample, and only two of these in more than two samples. Furthermore, there was no consistent association with parity or tumour phenotype (Table S7) and some recurrently mutated genes were identified in the sarcoma as well as epithelial tumours. These findings suggested that coding sequence mutations did not underlie the phenotypic changes seen in parous compared with virgin tumours.

Using the exome data to estimate copy number variations (CNVs) confirmed the qPCR analysis of the *Erbb2/Neu* locus in genomic DNA in eight of the nine tumours (Fig. 3B; Table S8 for detailed results, Table S9 for summary data). Four tumours that had a strongly amplified *Neu* locus by qPCR, all of which were AC(NST), showed amplification by exome data of a segment in chromosome 11 that included *Erbb2* but also nearby genes *Mien1* and *Grb7*. Two tumours also had amplified loci on chromosome 19, which included *Pten* and *Atad1*. In four tumours [two ASQCs, one an AC(NST) and a mammary sarcoma], all of which had low levels of amplification by qPCR, no CNVs passing the statistical threshold could be detected (Fig. 3B; Tables S8 and S9). Only one tumour, an AC(NST) (MS1218.2), showed discordant results between the qPCR and sequencing-based analysis of CNVs.

To broaden these findings to CNVs across a larger tumour sample, we carried out digital droplet PCR (ddPCR) for the genomic *Erbb2/Neu*, *Grb7*, *Mien1*, *Pten* and *Atad1* loci on the tumour cohorts and matched spleen samples, using both snap-frozen tumour samples and DNA isolated from formalin-fixed paraffin-embedded (FFPE) blocks. We also analysed the *NeoR loxP-stop-loxP* cassette to confirm its deletion (and thus activation of the *NeuKI* locus). We included *Erbb2/Neu* locus-specific probes to both the mutant *NeuKI* allele and exon 12 of the endogenous *Erbb2* gene (see schematic in Fig. S1). As controls, we used DNA extracted from primary mammary epithelial organoids isolated from 10-week-old virgin wild-type mice or from mice carrying the *NeuKI* allele but no CRE and cultured *in vitro* for 7 days.

As expected, in wild-type organoids, *NeoR* and *Neu* were undetectable, whereas *Erbb2* exon 12 was present in two copies. In *NeuKI* organoids, as expected, *NeoR* and *Neu* were each present in a single copy and *Erbb2* exon 12 was present in two copies. In most spleens from tumour cohort animals (Fig. S2A, see Table S10 for full numerical ddPCR results), *NeoR* and *Neu* were also present in a single copy, and *Erbb2* in two copies. This was as expected, given that *Blg* is considered a mammary-specific promoter. However, in one animal, MS1163, the *NeoR* cassette had been lost and *Neu* had undergone low level amplification. Therefore, there may be a low level of background recombination in the spleen, either due to leakiness of the *Blg* promoter or to non-specific effects of endogenous recombinases.

Twenty-six out of 29 tumours tested (of all phenotypes) showed evidence of *NeoR* recombination or locus amplification, or both (Fig. S2B-E). The exceptions were three ASQC tumours (Fig. S2D, samples MS1207-1 MS1349-1 and MS1448-1). When comparing tumours either by the parity status of the animals or the tumour histotype, there was broad agreement in the findings from the ddPCR with the CNV-by-exome analysis and the original genomic qPCR analysis of the *NeuNT/Erbb2* locus (Fig. 3C; Table S10). The mean copy number of the *Neu* allele was higher in tumours from virgin animals, and in tumours of the AC(NST) histotype. However, the range of amplification was wide, and these differences were significant by genomic qPCR but not significant by ddPCR for both comparisons. When considering individual tumours in which both ddPCR and qPCR had been performed on the *Neu* allele, there were discordant results for tumours MS1213-1 (virgin) and MS1218-2 (parous) (Fig. 3D). Both of these tumours had also been CNV profiled by exome sequencing (ExomeSeq), but in one case (MS1213-1) the ExomeSeq agreed with the qPCR result, while in the other (MS1281-2), the ExomeSeq agreed with the ddPCR result. It is not clear whether the differences arising from different approaches, and the discordant results between some individual tumours, are a result of technical limitations or from tumour heterogeneity leading to sampling biases. However, taken together, all the approaches used to analyse *NeuKI* allele copy number support the model that AC(NST) tumours tend to have higher levels of *NeuKI* amplification, whereas ASQC tumours have lower levels.

Confirming the CNV-by-exome analysis, ddPCR analysis of *Grb7* and *Mien1*, which are located close to *Erbb2* in both mouse and human (the locus is syntenic in the two species), demonstrated similar levels of amplification in the tumours to *Erbb2/Neu* (Fig. S3, Table S10). This supports a model in which the whole locus is a hotspot for amplification, and it is not simply a feature of the engineered allele. In contrast, although amplification of *Atad1* and *Pten*, as suggested by the CNV-by-exome analysis, was confirmed by ddPCR in some tumours, potential copy number losses were also observed in others (Fig. S4, Table S10). Thus, the significance of changes in *Pten* and *Atad1* is uncertain.

### *Erbb2*/*Neu* locus amplification is the prime determinant of tumour phenotype, and distinct gene expression patterns are associated with locus amplification state

Next, we performed RNA sequencing (RNAseq) analysis on the same tumours used for exome sequencing. Data were compared across the tumour sets as a whole to identify genes that were differentially expressed between biologically distinct tumour groups. Non-negative matrix factorization (see Materials and Methods) was used to compare samples on the basis of parity, tumour phenotype and *Erbb2/Neu* locus amplification status (by genomic qPCR) and to determine which of these comparisons generated the most distinct and stable sample clusters, i.e. which was the strongest driver of differences in gene expression pattern. The results showed that the strongest determinant of gene expression differences between the samples was amplification status of the *Erbb2/Neu* locus in the epithelial tumours, with the mammary sarcoma being an outlier (Figs S5 and S6A).

As amplification status was the strongest determinant of gene expression differences, the RNAseq data were analysed to determine which genes were significantly differentially expressed between high-amplified [*n*=5; all AC(NST)] and not/low-amplified [*n*=3; two ASQC and one AC(NST)] tumours (excluding the data from the sarcoma). Normalized fragments per kilobase per million fragments mapped (FPKM) values for each of these tumours are shown in Table S11 and the full list of genes significantly differentially expressed between the two classes is given in Table S12. Eight-hundred and sixty-five genes were significantly expressed >2-fold higher in high-amplified versus not/low-amplified tumours, with 619 genes being significantly >2-fold higher in the not/low-amplified tumours versus high-amplified tumours. Unsupervised hierarchical clustering of samples based on these significantly differentially expressed genes clustered the samples, as expected, by amplification status of the *Erbb2/Neu* locus (see heatmap in Fig. S6B). The six tumour samples with the highest levels of normalized *Erbb2* expression were from the six high-amplified tumours, while the three samples with the lowest levels of *Erbb2* expression were from the three not/low-amplified tumours.

### The EDC is activated in ASQC tumours

Functional annotation of differentially expressed genes was carried out using the DAVID (v6.8) on-line tool (Huang da et al., 2009a,b) using Gene Ontology (GO; Bioprocess) and Kyoto Encyclopedia of Genes and Genomes (KEGG) pathway analysis. Key GO/KEGG annotations of particular interest, together with their associated genes, are listed in Table 1, and include ERBB2 signalling and WNT signalling (see Table S13 for full dataset, with GO and KEGG results in separate tabs corresponding to analysis of gene sets differentially expressed in high-amplified and not/low-amplified tumours). Also of interest were two GO terms associated with keratinocytes (GO:0030216∼keratinocyte differentiation, GO:0031424∼keratinization), which were enriched in the not/low-amplified tumours, consistent with the ASQC histology of two of the three samples in this group. Keratinization is driven by activation of the EDC (Oh and de Guzman Strong, 2017), a group of co-regulated epidermal genes. To determine whether there was evidence for activation of the EDC in non/low amplified tumours/ASQCs, the genomic locations of each of the 1484 significantly differentially expressed genes from Table S12 were retrieved from the JAX Mouse Genome Informatics database, taking the chromosome on which the gene is located and its ‘start position’ as the genomic location (Table S14).

**Table 1.**
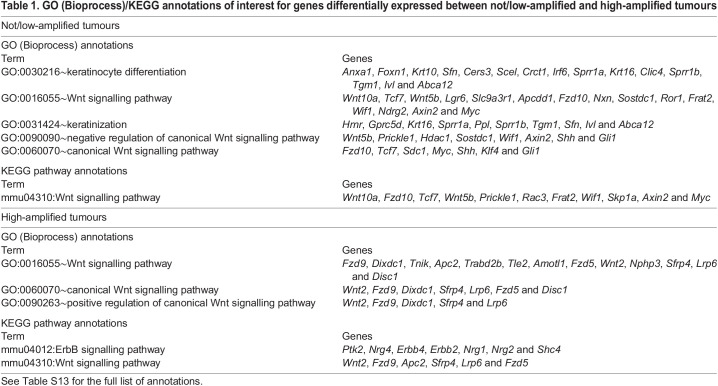
GO (Bioprocess)/KEGG annotations of interest for genes differentially expressed between not/low-amplified and high-amplified tumours

**Fig. 4.**
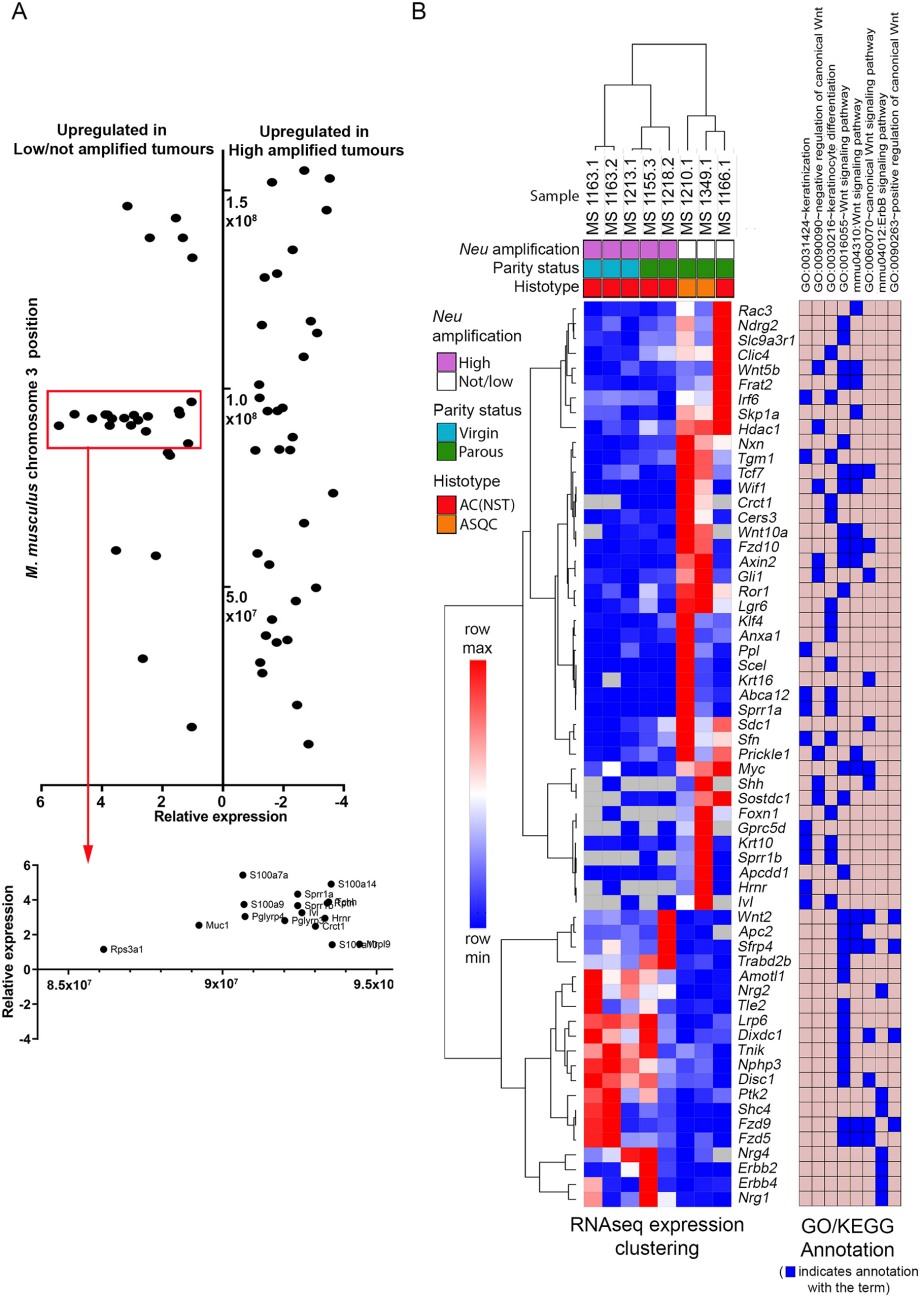
***Neu* amplification status is associated with differential WNT signalling and activation of the epidermal differentiation cluster.** (A) Log2 relative expression of genes on *M. musculus* chromosome 3 significantly differentially expressed between not/low-amplified and high-amplified tumours. Genes are plotted by their relative expression levels (*x*-axis) and their chromosomal location (*y*-axis) (Table S14). A cluster of genes is highly expressed in the not/low-amplified tumours immediately before base position 1×10^8^ (red rectangle), suggesting co-regulation. This region is expanded below and the genes annotated. This is the epidermal differentiation cluster, and includes genes associated with epithelial keratinization. (B) Unsupervised hierarchical clustering of Log2 FPKM data for genes of interest identified by functional annotation (mmu04310:Wnt signalling pathway, GO:0016055∼Wnt signalling pathway, GO:0060070∼canonical Wnt signalling pathway, GO:0090090∼negative regulation of canonical Wnt signalling pathway, GO:0090263∼positive regulation of canonical Wnt signalling pathway, mmu04012:ErbB signalling pathway, GO:0030216∼keratinocyte differentiation and GO:0031424∼keratinization) (see Table 1). *Neu* amplification status (by qPCR), parity, histotype are indicated. The GO/KEGG annotations of each gene are indicated.

In mice, the EDC is located on chromosome 3. Therefore, the relative expression levels of the differentially expressed genes located on chromosome 3 were plotted against their genomic locations (Fig. 4A). Notably, the genes on this chromosome that are upregulated in the not/low-amplified tumours, cluster to the EDC in a location just proximal to the 1×10^9^ base position, supporting the model that squamous metaplasia in ASQC tumours is driven by coordinated activation of the EDC.

### High- and not/low-amplified tumours express different patterns of WNT signalling-associated genes

The enrichment of WNT signalling-related KEGG/GO terms in the set of differentially expressed genes (Table 1) was of interest, as WNT signalling is an important regulator of mammary epithelial development (Jarde and Dale, 2012). Intriguingly, the annotations mmu04310:Wnt signalling pathway, GO:0016055∼Wnt signalling pathway and GO:0060070∼canonical Wnt signalling pathway were enriched in both the high- and not/low-amplified tumours, but the genes annotated by those two terms were different (Table 1)*.* To better understand WNT signalling-associated gene expression in the high- and not/low-amplified tumours, the tumour samples were analysed by unsupervised hierarchical clustering of the expression levels of the genes annotated by the mmu04310, GO:0016055, GO:0060070, GO:0090090 and GO:0090263 WNT-associated KEGG/GO terms. Also included in the analysis were the mmu04012:ErbB signalling pathway, GO:0030216∼keratinocyte differentiation and GO:0031424∼keratinization genes: a total of 61 genes.

This analysis (Fig. 4B) confirmed that the high- and not/low-amplified tumours had distinct patterns of expression of WNT-associated genes. Not only were different subsets of the mmu04310:Wnt signalling pathway, GO:0016055∼Wnt signalling pathway and GO:0060070∼canonical Wnt signalling pathway gene sets associated with the different tumour classes, but also genes annotated by the term GO:0090090∼negative regulation of canonical Wnt signalling pathway were associated with not/low-amplified tumours, while genes annotated by the term GO:0090263∼positive regulation of canonical Wnt signalling pathway were associated with high-amplified tumours. Importantly, repeating this analysis with the keratinocyte and ERBB2 pathway-associated genes excluded resulted in the same clustering pattern (Fig. S6C), confirming that the pattern of expression of WNT-associated genes does distinguish between the tumour classes.

### ASQC tumours have activated canonical WNT signalling

The WNT pathway-associated genes differentially expressed between not/low-amplified and high-amplified tumours suggest different branches of the WNT signalling pathway were being activated in these tumours. The two main WNT pathways are the canonical signalling pathway, characterized by the role of β-catenin as a nuclear transcription factor driving gene expression changes, and the non-canonical or planar cell polarity pathway, which regulates the actin cytoskeleton and is an important regulator of collective cell migration in tumour metastasis (VanderVorst et al., 2019). At face value, the association of GO:0090090 (negative regulation of canonical Wnt signalling pathway) with not/low-amplified tumours and GO:0090263 (positive regulation of canonical Wnt signalling pathway) with high-amplified tumours makes clear which pathway is active in each tumour class. However, regulation of WNT signalling pathways is highly complex. There are 19 mammalian WNT ligands interacting with one or more of ten mammalian FZD receptors (Najdi et al., 2012; Voloshanenko et al., 2017; Wang et al., 2016), and several co-receptors, as well as activation of feedback loops and interactions with other signalling pathways. WNT2 can activate both canonical and non-canonical WNT signalling (Mazzotta et al., 2016; Najdi et al., 2012), while FZD5 and FZD9, both of which are expressed in the high-amplified tumours, activate different branches of the pathway (FZD5 the canonical pathway and FZD9 the non-canonical pathway) (Wang et al., 2016). Furthermore, WIF1 (WNT-inhibitory factor 1) is a secreted inhibitor of canonical WNT signalling and, therefore, although annotated as a negative regulator of the pathway and found in the not/low-amplified tumours (Table S14), it is also a target gene of canonical WNT signalling, acting as part of a negative-feedback loop. Its expression indicates activity of the canonical pathway.

**Fig. 5.**
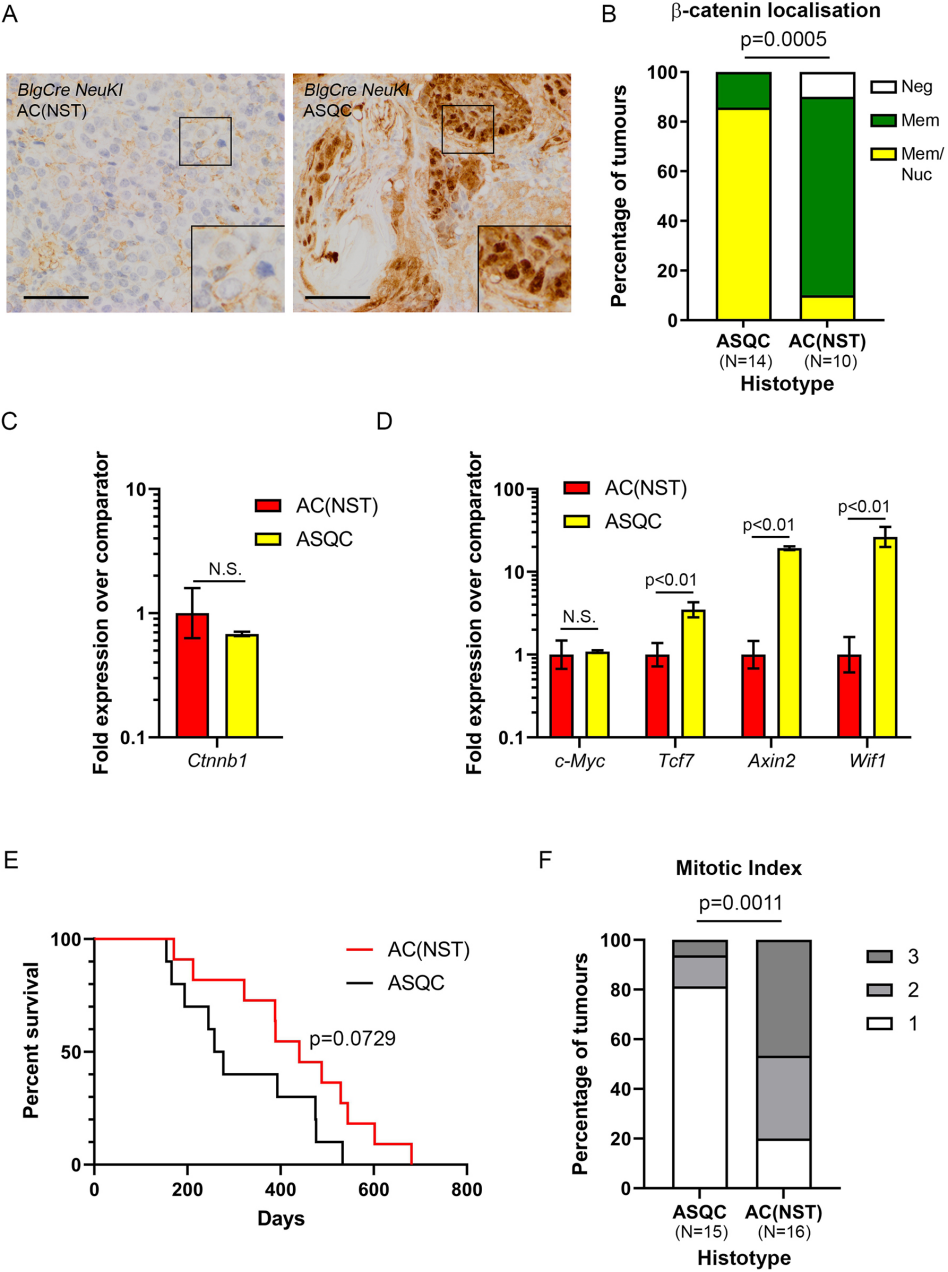
**Canonical WNT signalling is activated in ASQC tumours.** (A) Representative examples of membrane and nuclear β-catenin localization in AC(NST) and ASQC tumours. Scale bars: 50 µm. (B) Quantitation of β-catenin localization. *P*=0.0005 (Fisher's exact test of the proportion of tumours with nuclear staining versus no nuclear staining/no staining). (C) qrtPCR analysis of *Ctnnb1* gene expression [mean fold expression±95% confidence intervals relative to the AC(NST) cohort; *n*=8 AC(NST) and *n*=6 ASQC tumours, three technical replicates of each tumour; significance determined from confidence intervals (Cumming et al., 2007)]. (D) qrtPCR analysis of canonical WNT target gene (*Myc*, *Tcf7*, *Axin2* and *Wif1*) expression [mean fold expression±95% confidence intervals relative to the AC(NST) cohort as for C]. (E) Kaplan–Meier survival curves for *BlgCre-NeuKI* mice according to tumour histotype. Only mice with one or more ASQC tumours (*n*=10 animals), or one or more AC(NST) tumours (*n*=11 animals) were included. Mice with AME tumours, non-mammary epithelial tumours or that developed multiple tumours of different histotypes were excluded. *P*=0.0729 by Log-Rank test. (F) Comparison of mitotic index in 15 ASQC and 16 AC(NST) tumours from *BlgCre-NeuKI* mice. Fisher's exact test of proportion of tumours with grade 1 mitotic index versus tumours with grade 2 or 3 mitotic index as defined by the WHO breast cancer classification criteria (Lakhani et al., 2012). N.S., not significant.

Given this complexity, to directly determine in which tumour type canonical WNT signalling was active, we assessed nuclear β-catenin staining and measured expression of canonical WNT target genes [*Myc*, *Tcf7*, *Axin2* and *Wif1*] by quantitative real-time RT-PCR (qrtPCR) in AC(NST) and ASQC tumours of the *BlgCre-NeuKI* cohort. Nuclear β-catenin staining was highly significantly (*P*<0.001) associated with ASQC tumours (Fig. 5A,B), although there was no significant difference in levels of *Ctnnb1* gene transcription between the histotypes (Fig. 5C). Interestingly, the single AC(NST) tumour in which nuclear β-catenin staining was observed (MS1217-1) had relatively low levels of *Neu* amplification by qPCR (3.2-fold; Fig. 3A). There was no difference in *Myc* expression between AC(NST) and ASQC tumours; however, expression of *Tcf7*, *Axin2* and *Wif1* were all significantly (*P*<0.01) elevated in ASQC tumours (Fig. 5D; detailed results provided in Table S15). Canonical WNT signalling is, therefore, activated in the ASQC histotype and not in the AC(NST) histotype. The non-canonical pathway is likely activated in the latter.

To assess the significance of elevated expression of canonical WNT target genes in human HER2 breast cancer, the KM plotter resource (Nagy et al., 2018) was used to mine relapse-free survival data for unselected human breast cancers, for HER2-amplified breast cancers and for HER2-non-amplified breast cancers stratified according to *MYC*, *TCF7*, *AXIN2* and *WIF1* expression (Fig. S7). The results show that high expression of *TCF7*, *AXIN2* and *WIF1* all predict shorter relapse-free survival (RFS) in HER2-amplified breast cancer, but longer RFS in HER2-non-amplified disease. Interestingly, the pattern is reversed for *MYC* expression. Although RFS in human disease cannot be directly correlated with survival curves for mouse tumour models, these findings suggested that expression of canonical WNT target genes in the mouse tumours might be associated with differences in survival. We had already shown that parity in the *BlgCre-NeuKI* cohort was associated with a slightly shorter median survival compared with virgin animals, although this was of borderline significance (Fig. 1C). We had also shown that the ASQC phenotype (in which canonical WNT signalling is active) was associated with parity (Fig. 1F). Therefore, we directly tested whether animals that developed only ASQC phenotype tumours had a difference in survival compared with animals that developed only AC(NST) tumours. ASQC tumour-bearing animals had a shorter median survival of 267 days compared with AC(NST) animals with a median survival of 440 days (Fig. 5E), although this was not significant. However, these curves are based on the time at which mice had to be euthanized because the tumours reached specified size limits. They do not reflect growth rates of the tumours. Indeed, comparison of mitotic index scores, based on the World Health Organization (WHO) breast tumour grading guidelines, demonstrate that ASQC tumours had a significantly lower mitotic index than AC(NST) tumours (Fig. 5F). This suggests that ASQC tumours had an earlier onset than AC(NST) tumours but grew more slowly; therefore, the overall survival of the animals with the tumours was not significantly different.

Therefore, unlike our previous analyses of the sensitivity of different mammary epithelial cell populations to loss of *Brca1*, *Brca2*, *Pten* and *p53*, the basal mammary epithelial layer is less sensitive to transformation by activation of the *NeuKI* allele, whereas the luminal ER^−^ mammary epithelial cells are more sensitive. Activation of the allele in luminal cells in virgin animals tends to generate AC(NST) tumours associated with high locus amplification. In contrast, in parous animals, activation tends to generate ASQC tumours with lower levels of locus amplification and activation of both the EDC and canonical WNT signalling. ASQC tumours tend to occur earlier than AC(NST) tumours, but grow more slowly. The key features of the different models we describe here are summarized in Table 2.

**Table 2.**
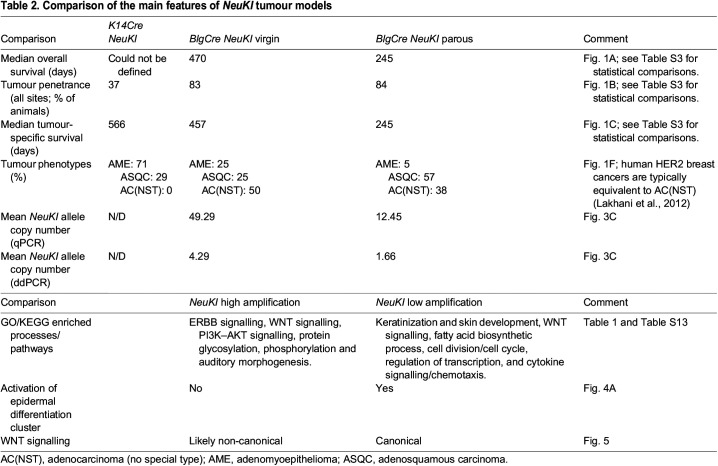
Comparison of the main features of *NeuKI* tumour models

## DISCUSSION

Use of *MMTV*-*Neu* mouse models has been key in identifying many of the genetic and molecular events that control *HER2*-induced breast cancer progression (Bouchard et al., 1989; Muller et al., 1988). Both wild-type (*NeuN*) and activated (*NeuNT*) forms of Neu have been used; however, both drive rapid tumour formation as a result of the activity of a strong exogenous promoter, rather than amplification of the genomic locus, as occurs in the human disease (Jacot et al., 2013). In contrast, when *NeuNT* was knocked into the endogenous *Erbb2* locus, and therefore expressed under the control of the endogenous promoter, *MMTV-Cre*-dependent activation of expression resulted in mammary hyperplasia and the formation of focal tumours after a long latency (over 1 year) (Andrechek et al., 2000). Importantly, tumour formation in this system was associated with *Erbb2/NeuNT* locus amplification (Andrechek et al., 2000) by an unknown mechanism. Tumours from the *MMTV-Cre NeuNT* Knock-In (*MMTV-Cre NeuKI*) mouse model showed similar additional genomic abnormalities to *HER2*-initiated human breast cancer (Hodgson et al., 2005; Montagna et al., 2002). However, the target cell for tumour formation in both *MMTV-NeuN/NT* and *MMTV-Cre NeuKI* models has not been defined, and likely depends on which mouse line is used. These models have been unable, therefore, to contribute to our understanding of how cell-of-origin may affect HER2 amplification, tumour formation, phenotype and behaviour in humans.

We have previously demonstrated that tumour phenotypic heterogeneity is driven by a combination of the cell-of-origin and initiating genetic lesion, and that the ER^−^ luminal stem/progenitor population has the potential to be the origin of both TNBC-like and ER positive-like mammary tumours. To determine whether cell-of-origin also affects development and phenotype of HER2-amplified tumours, we used our cell type-targeted promoter approach to activate the *NeuNT* allele in either basal or luminal ER^−^ mammary epithelial populations. In contrast to our previous studies in which *Brca1*, *Brca2*, *Pten* and *p53* were conditionally deleted in these populations (Melchor et al., 2014; Molyneux et al., 2010), we found that the basal mammary epithelium is less sensitive to transformation by *NeuKI* activation than the luminal epithelium. This demonstrates that at least one reason for distinct organ tumour tropisms associated with specific somatic or germline mutations is that individual cell types are differentially sensitive to transformation by different genetic lesions. The reason that luminal ER^−^ cells are sensitive to *NeuKI* activation is not clear, but we suggest it is, at least in part, due to the role of WNT signalling in mammary development/pregnancy and interactions between these two pathways.

During early mammary development and the ductal elongation, which occurs during puberty, the most highly expressed mammary WNTs are WNT2 (which can activate both canonical and non-canonical signalling) and WNT5A (non-canonical) (Weber-Hall et al., 1994), consistent with the role of non-canonical WNTs in planar cell polarity and the regulation of collective cell migration (VanderVorst et al., 2019). During pregnancy, there is a switch to the expression of canonical WNT4, WNT5B and WNT6 (Weber-Hall et al., 1994). The canonical pathway is typically associated with regulation of stem/progenitor cells, consistent with the activation of alveolar progenitors in preparation for milk production. We have previously demonstrated that strong activation of WNT signalling in organoid culture *in vitro* (by R-spondins) results in squamous metaplasia (Jarde et al., 2016). Here, our *in vivo* findings support this, with the ASQC tumour phenotype strongly associated with activation of canonical WNT signalling, triggered in this case by pregnancy. Pregnancy results in a spike of canonical WNT signalling within the mammary gland. Our findings suggest that, in the context of activation (but not amplification) of the *NeuKI* allele, this canonical WNT spike persists and becomes a chronic activity that leads to formation of tumours with squamous metaplasia. We suggest that in this system the canonical WNT and *NeuKI* allele-driven pathways are interacting at the level of β-catenin and Forkhead (FoxO) factors.

Transcription factors of the FoxO class have been characterized as positive transcriptional regulators of pro-apoptotic and cell cycle arrest genes (Stahl et al., 2002). In response to activation of PI3K–AKT signalling, nuclear FoxOs are phosphorylated and translocated out of the nucleus. Apoptosis is thus suppressed and cell cycle inhibition relieved, allowing proliferation. We propose a model in which activation of PI3K–AKT signalling by the *NeuNT* allele in virgin mice results in FoxO phosphorylation, translocation out of the nucleus, an increase in proliferation and protection from apoptosis. The stronger the NeuNT–PI3K–AKT signal, the more FoxO is excluded from the nucleus and the greater the increase in proliferation and protection from apoptosis. Hence, in a population of transformed cells, the greatest ‘fitness’ will be conferred on the clone with the strongest activity of the NeuNT–PI3K–AKT pathway. Therefore, clones in which the *NeuNT* locus has been amplified will emerge and dominate the tumour, as seen in virgin/amplified/AC(NST) tumours.

WNT and FoxO signalling are normally considered mutually inhibitory antagonistic pathways. For example, canonical WNT pathway activity shifts FOXO1 from a nuclear to a cytoplasmic location in an AKT-dependent manner, although how AKT is activated by canonical ligand activity remains unclear (Sreekumar et al., 2017). However, in colon cancer it has been reported that nuclear β-catenin and nuclear FoxO cooperate to promote tumour invasion and metastasis. Indeed, it was observed that if AKT activity was inhibited pharmacologically, the resultant increase in nuclear FoxO protein promoted cell invasion. Thus, in the context of an active canonical WNT signal, nuclear FoxO can switch from being a tumour-suppressing factor to a tumour-promoting factor. Therefore, the genomic variations observed in our mouse model are likely to result from changes in selection pressure on clonal populations arising from differences in the order in which WNT and NeuNT signalling are activated. If strong canonical WNT signalling occurs (e.g. during pregnancy) when NeuNT has been activated but prior to expansion of a *NeuNT*-amplified clone, this would allow tumour-promoting levels of β-catenin and FoxO to accumulate in the nucleus. In this case, if *Neu* were amplified it would boost the PI3K–AKT signal, drive FoxO out of the nucleus and, in the context of canonical WNT signalling, decrease fitness. Thus, in this context, expansion of a highly amplified clone would be selected against. Assessment of a time course of *Erbb2/Neu* expression and amplification as the luminal mammary epithelium progresses from normal, through preneoplasia to the neoplastic state, in both virgin and parous animals, would enable these processes to be better delineated.

Although we suggest that the emergence of *NeuNT* amplified or not/low-amplified clones depends on the outcome of interaction between WNT and FoxO signalling, it is unclear from our findings whether the WNT signal is itself directly driving locus amplification. *Erbb2/Neu* amplification is likely a result of oncogene-induced replicative stress. This is characterized by inappropriate replication origin licencing/firing leading to collisions between the replication fork and transcriptional machinery, resulting in replication fork stalling and collapse, and the formation of double-stranded DNA breaks (Hills and Diffley, 2014). This increase in genomic instability results in junctions forming between chromosomes, which resolve during cell division to create ‘neochromosomes’ – so-called chromoanasynthesis events – that are susceptible to focal amplification. Chromoanasynthesis has been demonstrated to be a mechanism of *ERBB2* amplification (Vasmatzis et al., 2018).

A distinct feature of ASQC tumours is activation of the EDC, a group of co-regulated genes for proteins required for keratinization of skin cells to form the protective barrier of the epidermis. Co-regulated gene clusters are typically under the control of ‘super-enhancer’ genetic elements and an H3K27ac chromatin immunoprecipitation with sequencing (ChIP-seq)-based catalogue identified super-enhancers associated with epidermal development in the mammary epithelium (Hnisz et al., 2013). In keratinocyte precursors and differentiated keratinocytes, super-enhancer elements are highly enriched in ΔNp63-binding sites (Cavazza et al., 2016). ΔNp63 is a key transcriptional regulator typically expressed in basal layers of stratified epithelia, such as the epidermis, the prostatic epithelium, the myoepithelial cells of the mammary gland and the basal-like cell compartment, which is seen in AME phenotype tumours. Expression of ΔNp63 is regulated by a complex network of well-known developmental pathways (in particular NOTCH, canonical WNT, Hedgehog, FGFR2 and EGFR signalling), often with complex negative- and positive-feedback loops, which are characteristic of systems that establish and maintain tissue boundaries (reviewed by Yoh and Prywes, 2015). ΔNp63 expression is one of the diagnostic features of metaplastic adenosquamous tumours in the mouse and also in the human (Tse et al., 2006), with strong ΔNp63 nuclear positivity seen in regions undergoing squamous metaplasia. Thus, we suggest that, in our system, canonical WNT and ERBB2 pathway activation combine to strongly activate ΔNp63, which in turn activates the EDC and drives the squamous metaplastic phenotype. It may be that a lower level of ΔNp63 activity drives cells into a basal epithelial lineage without activation of the EDC, instead resulting in the formation of an adenomyoepithelioma. Differences in the regulation of ΔNp63 between the mammary epithelium of the mouse and human may underlie the differences in frequency of breast tumours with adenomyoepithelial and adenosquamous features between the two species.

Although adenosquamous tumours of the breast are rare in humans, tumours with a squamous histotype are common in other body sites, e.g. the lung, prostate, pancreas and skin. Notably, dysregulated WNT signalling has been previously linked with overproliferative skin diseases of humans such as psoriasis (Gudjonsson et al., 2010), and activating WNT pathway mutations are important tumour drivers in human squamous cell carcinoma (SCC) (Lee et al., 2014). Furthermore, upregulation of putative WNT transcriptional targets has been demonstrated in feline oral SCCs (Giuliano et al., 2016) and the murine SCC model is WNT driven (Malanchi et al., 2008). Therefore, it is likely that the WNT–ΔNp63–EDC–squamous metaplasia pathway is common across cancers and that tumours with a squamous phenotype should be considered for therapy that targets canonical WNT signalling. Notably, a recent study found that 44% of breast cancers showing squamous metaplasia had WNT pathway mutations, compared with 28% of triple-negative breast cancers of no special type (Piscuoglio et al., 2017).

In human HER2-non-amplified tumours, high expression of canonical WNT target genes was associated with better prognosis. This is consistent with the less aggressive phenotype of the non-*NeuKI* amplified, canonical WNT-active ASQC mouse tumours. Metaplastic adenosquamous human breast tumours, as a subset of triple-negative breast cancer, would be expected to fall within the HER2-non-amplified tumour definition, although their overall rarity means they would only make up a very small proportion of that group. In contrast, canonical WNT signalling was associated with more aggressive tumours in HER2-amplified disease in humans, whereas canonical WNT signalling was not upregulated in the HER2-amplified mouse tumours. This difference highlights the caveats of comparing mouse and human tumours, and the importance of recognizing that there are likely to be both similarities and differences in the fundamental biological mechanisms driving tumour formation in the two species.

The formation of sarcomas, both in the mammary gland and on the head/neck, was a unique feature of this model. The *BlgCre* allele used here was the same as we have previously used, with no evidence of any tumour formation outside the mammary gland until now. However, while *BlgCre* mainly targets the luminal ER− population (Molyneux et al., 2010), we cannot exclude low-level off-target activation in other tissues. If a particular off-target cell type is also particularly sensitive to the genetic lesion being induced in this manner, then a low-level background of tumours from other tissues will be seen. Potentially, mammary and head/neck mesenchymal cells are sensitive to activation of the *NeuKI* allele, but not to the other alleles we have previously used. Importantly, however, the *K14Cre* and *BlgCre* drivers we have used here are the same ones we have used in previous studies and therefore our results – in particular the difference in *K14Cre*-driven cohorts – are comparable.

Our study has limitations. No virgin ASQC samples were available for analysis of *Erbb2/Neu* amplification by qPCR for direct comparison to parous ASQC and virgin/parous AC(NST) samples (Fig. 3). We were however able to analyse amplification from three virgin ASQC samples by ddPCR (Fig. S2). For FFPE samples analysed by ddPCR, there is the potential that analysis of nucleic acids, which have been through FFPE processing and subsequent extraction, may be prone to artefacts. However, FFPE samples were likely to contain a high percentage of viable tumour cells (following H&E histology), although we do not know what percentage of tumour cells was in a piece of snap-frozen tissue. Unfortunately, no samples were available in which a direct comparison of analysis of *Erbb2* locus amplification had been carried out by all four approaches available to us: qPCR (snap-frozen samples), CNV-by-exome (snap-frozen samples), ddPCR (snap-frozen samples) and ddPCR (FFPE samples). Without this, it is difficult to determine whether any one approach is a source of systematic technical errors or sampling variation. These caveats must be considered when interpreting our results. Nevertheless, Fig. 3D shows that, for most samples that we were able to test using multiple methods (although all from snap-frozen tissue), the conclusions on *Erbb2/Neu* amplification were in broad agreement.

In summary, targeting of *Erbb2/Neu* activation to specific cell populations within the mouse mammary epithelium supports the role of luminal ER^−^ stem/progenitor cells as the cells of origin for most breast cancer subtypes: TNBC/‘basal-like’, luminal ER^+^ (Melchor et al., 2014; Molyneux et al., 2010) and now HER2^+^. In this model, reproductive history, *Erbb2/Neu* activation and WNT signalling all interact to drive tumour phenotype, and a key determinant of that phenotype is the activation status of the ΔNp63-regulated EDC, which underlies squamous metaplasia. These results add reproductive history to cell-of-origin and initiating genetic lesion as interacting factors that determine mammary tumour phenotypic heterogeneity. Importantly, our findings also suggest that cell type-specific intrinsic sensitivity towards the transforming potential of activated oncogenes (and potential tumour suppressor genes) is at least one mechanism underlying the propensity for different mutations to generate tumours in a specific range of tissues.

## MATERIALS AND METHODS

### Experimental mice

All procedures were conducted according to UK Home Office regulations and Animal Research: Reporting of *In Vivo* Experiments (ARRIVE) guidelines, and under the authority of appropriate licences following local ethical review by the Cardiff University Animal Welfare Ethical Review Body. Mice carrying the targeted *NeuKI* allele (see schematic in Fig. S1A) were kindly supplied by Prof. Margaret Frame (University of Edinburgh, UK) with permission from the originator (Prof. Bill Muller, McGill University, Montreal, Canada). Mice of two genotypes, *Krt14Cre-NeuKI* and *BlgCre-NeuKI*, were bred and maintained on a mixed FVB/C57Bl6 background. Some animals from each genotype were aged as virgin animals, others went through one or more pregnancies (full details of all animals used are provided in Table S1). *Cre* and *NeuKI* alleles were maintained as heterozygous loci; the non-recombined *NeuKI* locus is inactive and homozygosity of this allele is lethal. Genotyping primers and conditions are given in Table S2.

The well-established *MMTV-NeuNDL* mouse line (a kind gift from Don White, Institute of Cancer Research, London) (Siegel et al., 1999) was maintained to enable comparison with a widely used model of *HER2* disease driven purely by strong overexpression. For analysis of gene expression in wild-type mice, C57Bl6 animals were used. Wild-type and *MMTV-NeuNDL* mice have two functional endogenous *Erbb2* alleles, and the *MMTV-NeuNDL* mice have, in addition, rat-derived *Neu* transgenes driven by the *MMTV* promoter. In contrast, *Krt14Cre-NeuKI* and *BlgCre-NeuKI* mice have only a single functional endogenous *Erbb2* gene, as well as a single copy of the engineered *Neu* oncogene, expression of which is driven by the endogenous promoter but only when the *neo* STOP cassette is recombined (Fig. S1A). As the *NeuKI* allele is knocked into the endogenous *Erbb2* locus, once activated by CRE-dependent recombination of the *Flox-STOP-Flox* cassette, it is expressed under the control of the endogenous *Erbb2* promoter at physiologically comparable levels. It is not directly regulated by the *K14* or *Blg* promoters. In contrast, in the *MMTV-NeuNDL* mouse, *NeuNDL* is directly regulated by the strong *MMTV* promoter.

This study took place over an extended period of time, with samples being collected for the *NeuKI* cohorts between 2011 and 2015. Censored *K14Cre-NeuKI* animals were born later in this period, and we cannot definitively exclude genetic drift occurring in these mice over that time scale. It should be noted, however, that the *BlgCre* cohorts included animals born from 2010 to 2014; there was no obvious pattern to which animals in these cohorts developed tumours and which did not and were therefore censored.

### Flow cytometric isolation of mammary subpopulations

Single cells were prepared from fourth mammary fat pads of 10-week-old virgin female mice as described previously (Regan et al., 2012) and stained using anti-CD24-FITC (clone M/69 at 1.0 µg/ml; BD Biosciences; catalogue #553261), anti-Sca-1-PE (clone D7 at 1.0 µg/ml; eBioscience; catalogue #17-5981), anti-CD49f-PE-Cy5 (clone GoH3 at 5.0 µl/ml; BD Biosciences; catalogue #551129) and DAPI. Single-stained samples were used to set compensation values and establish cut-offs to define positive/negative populations. Basal mammary stem cells, basal non-stem cells, luminal ER^−^ cells and luminal ER^+^ cells were gated as previously described (Regan et al., 2012). Endogenous *Erbb2* expression in the different populations was analysed using a Taqman qrtPCR probe specific for the mouse transcript (Table S2).

### Histology and immunohistochemistry

Tumour tissue was fixed in ice-cold 4% neutral buffered formalin for 24 h before being processed into paraffin blocks according to standard procedures. Tissue sections (5 μm) were either stained using H&E for histological analysis, or were used for immunohistochemistry as described previously (Melchor et al., 2014). The following antibodies were used: anti-K14 (ab7800; 1:500; Abcam), anti-K18 (65028; 1/5; Progen Biotechnik), anti-ΔNp63 (ab735; 1:100; Abcam), anti-ERα (VP-E613; 1:500; Vector Labs), anti-PRA (hPAa7; 1:500; ThermoFisher Scientific), anti-PRB (αPR6; 1:75; Abcam), anti-β-catenin (clone 14; 1:200; BD Biosciences), anti-ERBB2/Neu (Ab-3 3B5 OP15; 1/500; Calbiochem) and anti-ERBB3 (B-3 Ab-7 2C3 MS-313-P0; 1:50; Neomarkers). Mouse tumour phenotyping, based on H&E appearance and on levels and staining patterns of ΔNp63, keratins, ER and progesterone receptor (PR), was carried out as previously described (Melchor et al., 2014; Molyneux et al., 2010) according to the WHO classification of tumours in the breast (Lakhani et al., 2012) by M.J.S. with support and advice from Professor Barry Gusterson (FRCPath, University of Glasgow, Western Infirmary, Glasgow).

### qrtPCR

Total RNA was isolated from tissues using Trizol (Life Technologies) or the RNeasy kit (QIAGEN) according to the manufacturer's protocol. One microgram of RNA was reverse transcribed using Quantitect (Life Technologies) according to the manufacturer's protocol. Gene expression analysis was carried out using TaqMan Universal PCR Mastermix according to the manufacturer's protocol (Applied Biosystems, Life Technologies). TaqMan probes (Table S2) were obtained from Taqman gene expression assays (Applied Biosystems). Data analysis was carried out using QuantStudio 7 (Applied Biosystems). Relative expression levels of target genes were calculated using the ΔΔC_t_ method as described previously (Kendrick et al., 2008) with *Actb*, *B2m* and *Gapdh* as the endogenous controls.

### ddPCR for CNV

DNA for ddPCR was extracted using the Gentra Puregene Tissue Kit (QIAGEN) as per the manufacturer's guidelines. Test samples were diluted to 300 ng/µl in RNase/DNase-free water, and CNV assays were prepared for partitioning by adding 300 ng of DNA per CNV assay probe (Table S2) to a restriction enzyme mix containing 0.01 U each of PacI, PsiI and MluI. Samples were incubated for 5 min at 37°C before chilling and addition of ddPCR buffer and reference probe. This mix was then aliquoted into PCR strips containing individual test probes and RNase/DNAse-free water to a total of 25 µl per sample. The partition mix was then transferred into the appropriate wells of a Bio-Rad droplet generation cartridge, followed by addition of droplet oil to respective wells, the cartridge gasket sealed and droplets generated using the Bio-Rad QX200 Droplet Generator. Droplets were subsequently transferred to a PCR plate for amplification consisting of one initial cycle of 95°C for 10 min followed by 40 cycles of 94°C for 30 s and 60°C for 1 min with a final denaturing step of 98°C for 10 min. Plates were then analysed within 1 h of droplet generation on a Bio-Rad QX200 droplet reader. Analysis was performed using the QuantaSoft Analysis program (Bio-Rad).

### Next-generation sequencing analysis

Ten *BlgCre-NeuKI* tumours with representative phenotypes were selected for RNAseq-based analysis of gene expression. Genomic DNA was isolated from the same ten tumours, together with matched spleens, for exome sequencing. Next-generation sequencing analysis was carried out by the Institute of Cancer Research Tumour Profiling Unit (Chester Beatty Laboratories, London, UK).

For RNAseq, genomic DNA was removed from 500 ng of total RNA using the genomic DNA eliminator column from RNeasy Plus Micro Kit (QIAGEN) and rRNA removed using RiboZero (Epicentre) following the manufacturer's instructions. Strand-specific libraries were created using NEBNext Ultra Directional RNA Library Prep Kit for Illumina and 20 ng of the rRNA-depleted RNA.

For ExomeSeq, genomic DNA (200-600 ng) was fragmented to 200 bp using a Covaris E Series and the resultant libraries were subjected to DNA Capture using SureSelect XT Mouse All Exon kit (Agilent, Stockport, Cheshire) following the manufacturer's instructions.

Final libraries from both RNAseq and ExomeSeq preparations were quantified using qPCR and clustered at a molarity of 14.5 pM; sequencing was performed on an Illumina HiSeq 2500 using 2×76 cycles of version 3 SBS chemistry. Raw and processed RNAseq and ExomeSeq data have been deposited at Gene Expression Omnibus (GEO) with the overall accession number GSE162348.

### Bioinformatic analysis of RNAseq data

Raw FASTQ sequence files were quality control checked using FastQC (http://www.bioinformatics.babraham.ac.uk/projects/fastqc). To estimate gene expression, reads were aligned to the mouse genome (build 38) using StarAlign (Dobin et al., 2013) with no more than three mismatches, and only uniquely mapped reads were permitted. Reads for which ratio of mismatches to mapped length was greater than 0.10 were also discarded. All other parameters were set to their defaults for unstranded alignment. The expression level, based on FPKM, of each gene present in the mouse GTF annotation (build 38) file downloaded from Ensembl (Yates et al., 2016) was estimated using Cufflinks (Trapnell et al., 2010) with library type defined as ‘fr-unstranded’. All other parameters were set to defaults. Read counts across each gene were calculated by HTSeq-count (Anders et al., 2015), and input to DESeq2 (Love et al., 2014) to detect differential expression. FPKMs from multiple samples were merged to generate a gene-by-sample matrix using a custom Perl script for input into downstream signature scoring algorithms.

Non-negative matrix factorization (NMF) was used to cluster tumour gene expression data. Only the most highly expressed and variable genes were chosen for clustering according to the following criteria: (mean FPKM+SD)>1.00 and CV>0.10, where CV=coefficient of variation. The underlying principle of NMF is dimensionality reduction in which a small number of meta-genes, each defined as a positive linear combination of the genes in the expression data, are identified and then used to group samples into clusters based on the gene expression pattern of the samples as positive linear combinations of these meta-genes. Using the R package NMF (Gaujoux and Seoighe, 2010), factorization rank *k* was chosen by computing the clustering for *k*=2-6 against 50 random initializations of both the actual and a permuted gene expression matrix, and selecting the *k* value achieving the largest difference between cophenetic correlation coefficients calculated from the actual and permutated data. For further visual confirmation of a sensible choice of *k*, consensus matrices were generated corresponding to different *k* values. To achieve stability, the NMF algorithm was then run against 200 perturbations of each gene expression matrix at the chosen value of *k*=3. With the exception of sample MS1218.2, ESTIMATE analysis (Yoshihara et al., 2013) of the RNAseq data demonstrated that the samples had greater than 80% purity of tumour cells. Heatmaps were produced using the Broad Institute Morpheus tool, with default parameters for unsupervised hierarchical clustering.

### Mutation calling in ExomeSeq data

Read pairs were mapped against the mouse genome (build 38) using BWA ‘mem’ algorithm with default parameters (Li and Durbin, 2009). The resulting bam files were then pre-processed in preparation for somatic mutation detection using the Genome Analysis Toolkit (GATK) v3.5 best practice pipeline (Van der Auwera et al., 2013) and dbsnp version 144 in the base recalibration step (Sherry et al., 2001). MuTect v1.1.7 was then applied to compare the resulting bam files from tumour and matched normal tissue with call somatic mutations (Cibulskis et al., 2013). Mutations were annotated using the Ensembl Variant Effect Predictor (McLaren et al., 2016) using the canonical transcript, and non-silent protein coding mutations taken forward for further consideration.

### Copy number profiling using ExomeSeq data

Read pairs were mapped against the mouse genome (build 38) using BWA ‘mem’ algorithm with default parameters (Li and Durbin, 2009). Duplicate reads were removed, as were reads achieving mapping quality below 37. Depth of coverage at each position of all exons annotated according to the UCSC Genome Browser was calculated using GATK ‘DepthOfCoverage’ tool (Van der Auwera et al., 2013), and the resulting tumour and normal profiles input to ExomeCNV R package using default parameters (Sathirapongsasuti et al., 2011). Gene level log2 copy number ratios were then parsed using custom Perl scripts, with gains achieving log2 ratio >4.00, and losses <−2.50 taken forward for further consideration.

### Statistics

Statistical analysis was carried out in GraphPad Prism. Significance of survival curves was determined using Log Rank Mantel–Cox test and the Gehan–Breslow–Wilcoxon test. Statistics for differences between tumour numbers per animal used the Mann–Whitney test. Comparison of *Neu* amplification status used two-tailed *t*-test with Welch's correction on Log10-transformed values. Significance of gene expression differences analysed by qrtPCR were determined from 95% confidence intervals (Cumming et al., 2007).

## Supplementary Material

Supplementary information
