# Supplementary material for: Reproductive history determines Erbb2 locus amplification, WNT signalling and tumour phenotype in a murine breast cancer model
Source: Dis Model Mech. 2021 May 18;14(5):dmm048736. doi: 10.1242/dmm.048736 (PMC8188886; doi:10.1242/dmm.048736)
Supplement: Supplementary information [file dmm-14-048736-s1.pdf]

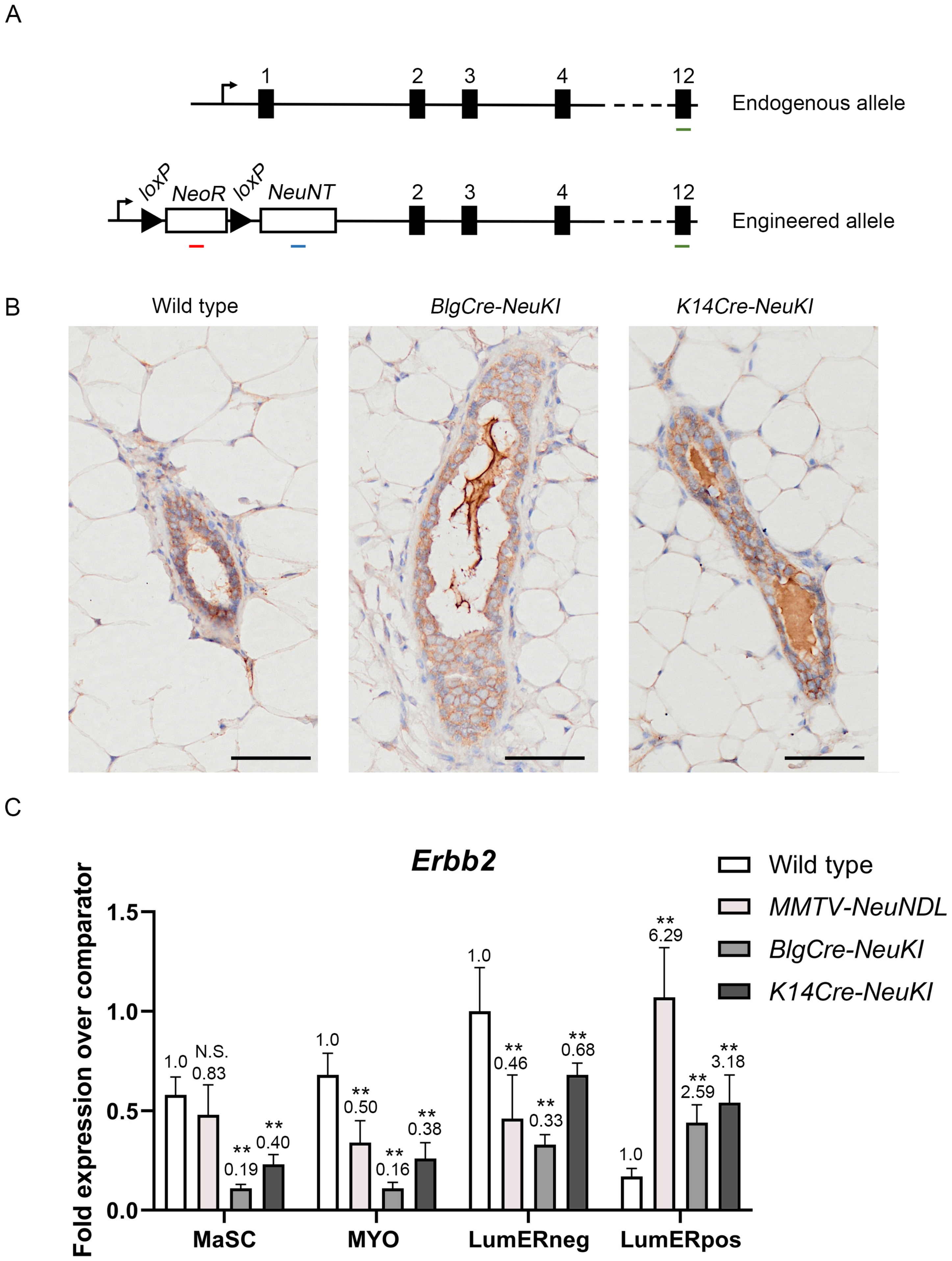

Disease Models & Mechanisms • Supplementary information

Figure S1: Schematic of *Erbb2* locus in *NeuKI* mice. (A) Modified from Andrechek et al., 2000, and indicating structure of wild type and engineered alleles and location of binding regions for ddPCR probes to the endogenous Erbb2 sequences (exon 12, green), the activated rat *NeuNT* sequence (blue) and the *loxP* – *NeoR* – *loxP* (STOP) cassette (red). **(B)** Staining for ERBB2 in mammary epithelium of 10-week-old wild-type, *BlgCre-NeuK* and *Krt14Cre-NeuKI* and mice. Bar = 50μm. **(C)** Comparison of *Erbb2* expression levels by mammary epithelial population across genetically modified mouse lines. Expression of endogenous *Erbb2* by qrtPCR in mammary epithelial subpopulations from 12-week-old wild-type, *MMTV-NeuNDL*, *Krt14Cre-NeuKI* and *BlgCre-NeuKI* mice. Data presented as relative expression ±95% confidence intervals relative to a comparator population (the luminal ER- cells from the wild type mice). Numbers above each column indicate the relative expression levels within each population compared to wild type mouse cells of the same population. For example, the expression of *Erbb2* in myoepithelial cells of *BlgCre-NeuKI* mice is 0.16 that of the expression of *Erbb2* in myoepithelial cells of wild type mice. **P<0.01 relative to expression levels of that population from wild type mice (determined from 95% confidence intervals) (Cumming et al., 2007). All data from three independent flow cytometric isolations, each of which was from pooled tissue of a minimum of 5 animals. MaSC, basal mammary stem cells; MYO, myoepithelial cells; LumERneg, luminal ER- progenitors; LumERpos, luminal ER positive differentiated cells.

Disease Models & Mechanisms • Supplementary information

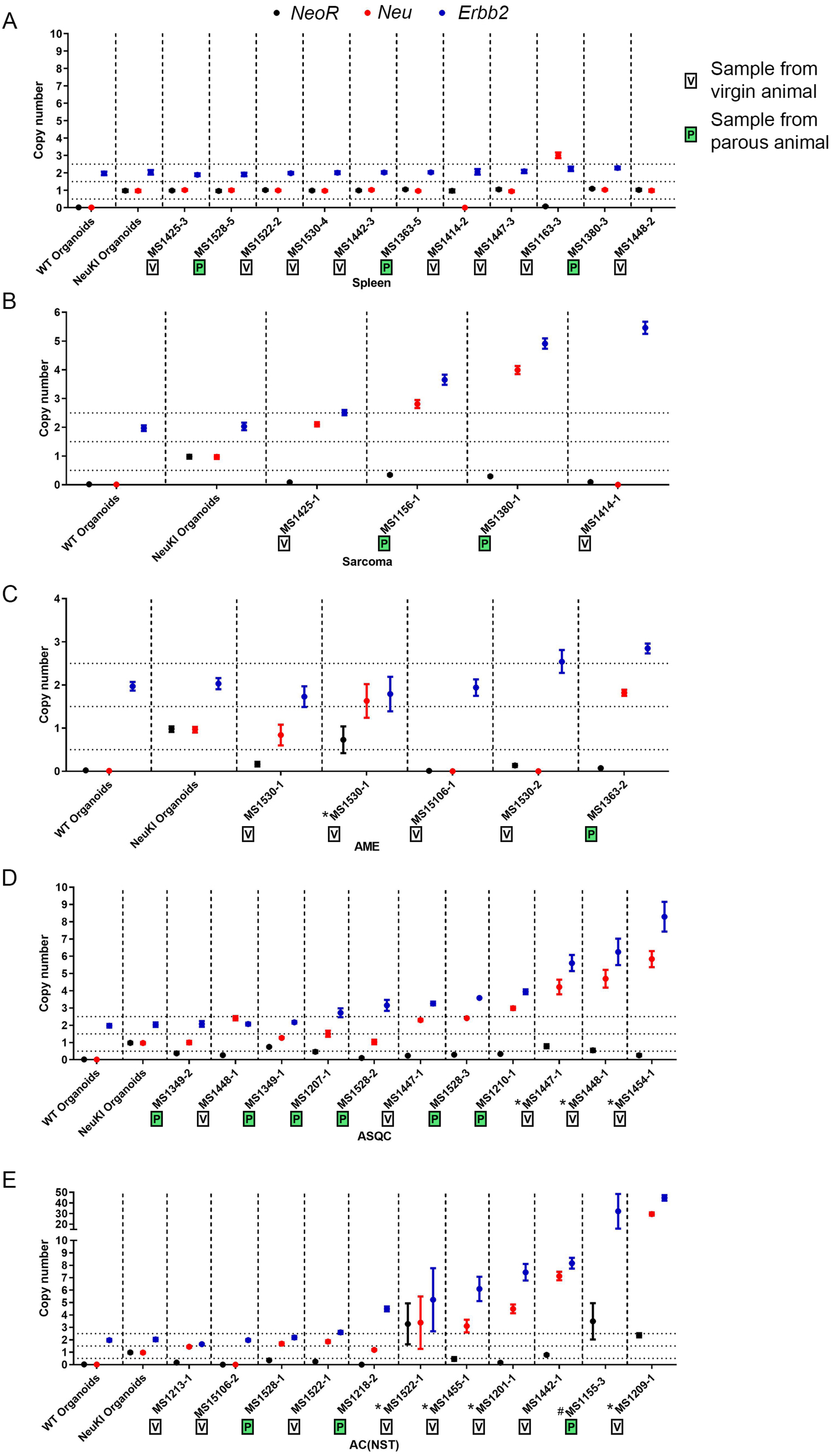

Disease Models & Mechanisms • Supplementary information

Figure S2: Amplification of both *Neu* and endogenous *Erbb2* alleles in *BlgCre-NeuKI* tumours. (A – E) Copy number of *NeoR* cassette, *Neu* and *Erbb2* alleles (mean±95% confidence intervals of three technical repeats for each sample) in spleens and tumour samples from *BlgCre-NeuKI* mice and in wild type and *NeuKI* organoid controls (eight biological replicates each of three technical replicates) by ddPCR. Samples are arranged from lowest to highest amplification from left to right. Black, *NeoR*; red, *Neu*; blue, *Erbb2*. Vertical dotted lines indicate the set of three results from each tumour sample. Horizontal lines are set at 0.5, 1.5 and 2.5. The non-recombined, non-amplified level for *NeoR* and *Neu* should be 1 (between the 0.5 and 1.5 marks); the level for *Erbb2* should be 2 (between the 1.5 and 2.5 marks). Parity status of animals is indicated. **(A)** Spleen. **(B)** Sarcomas. **(C)** AMEs. **(D)** ASQCs. **(E)** AC(NSTs). [#]The *Neu* ddPCR for MS1155-3 failed to be consistent, giving widely varying and often very high values. This result has been excluded. *Indicates analysis carried out on DNA extracted from FFPE material. For four tumours, both snap frozen and FFPE material were tested. See **Table S10** for detailed results.

Disease Models & Mechanisms • Supplementary information

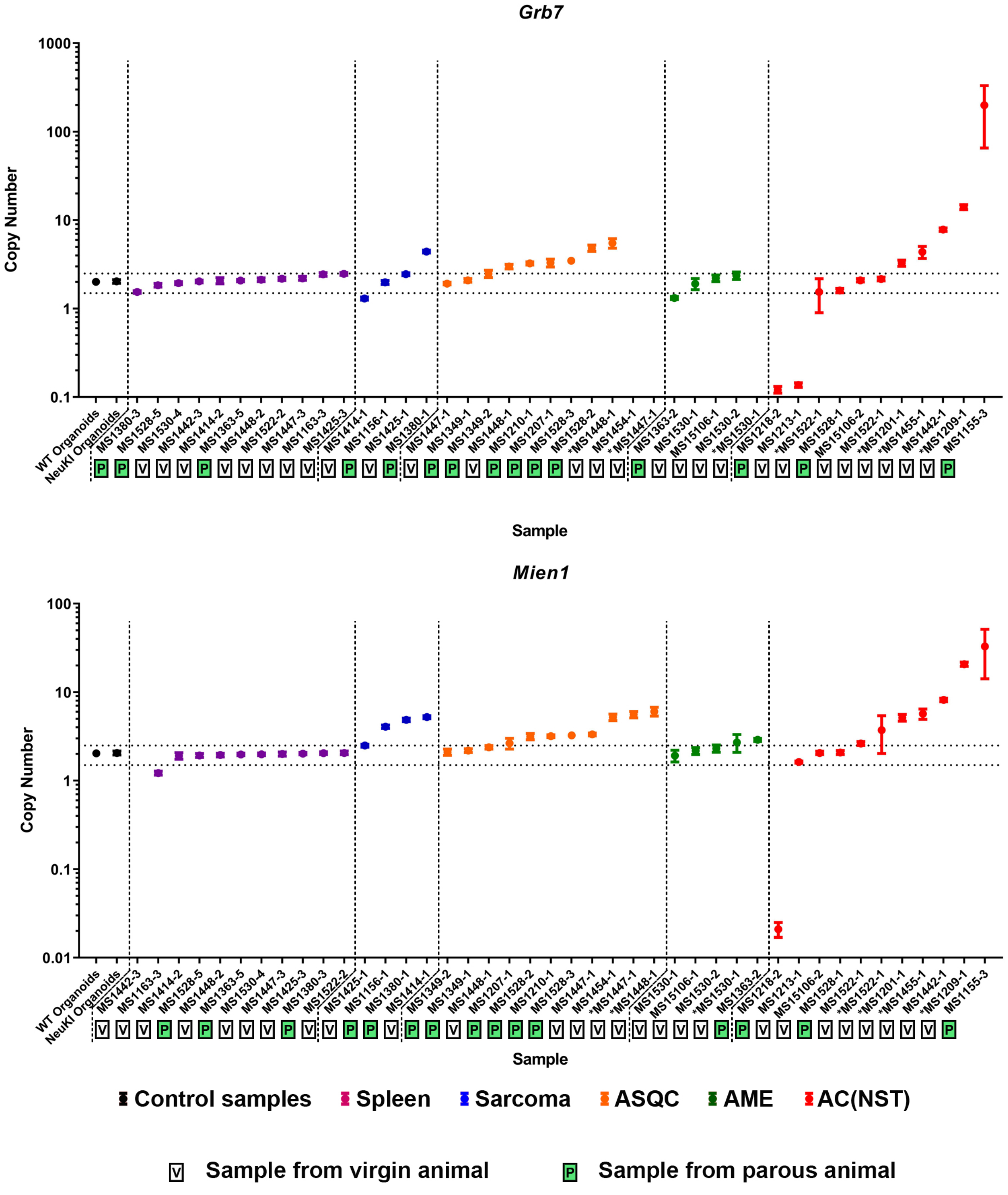

Disease Models & Mechanisms • Supplementary information

Figure S3: Copy number changes in *Grb7* and *Mien1* in *BlgCre-NeuKI* tumours. Copy number of *Grb7* (top) and *Mien1* (bottom) (mean±95% confidence intervals of three technical repeats for each sample) in spleens and tumour samples from *BlgCre-NeuKI* mice and in wild type and *NeuKI* organoid controls (eight biological replicates each of three technical replicates) by ddPCR. Samples are arranged from lowest to highest amplification from left to right and colour coded to indicate sample type (Black, control; purple, spleen; blue, sarcoma; yellow, ASQC; green, AME; red, AC(NST)). *Indicates analysis carried out on DNA extracted from FFPE material. For some tumours, both snap frozen and FFPE material were tested. See **Table S10** for detailed results.

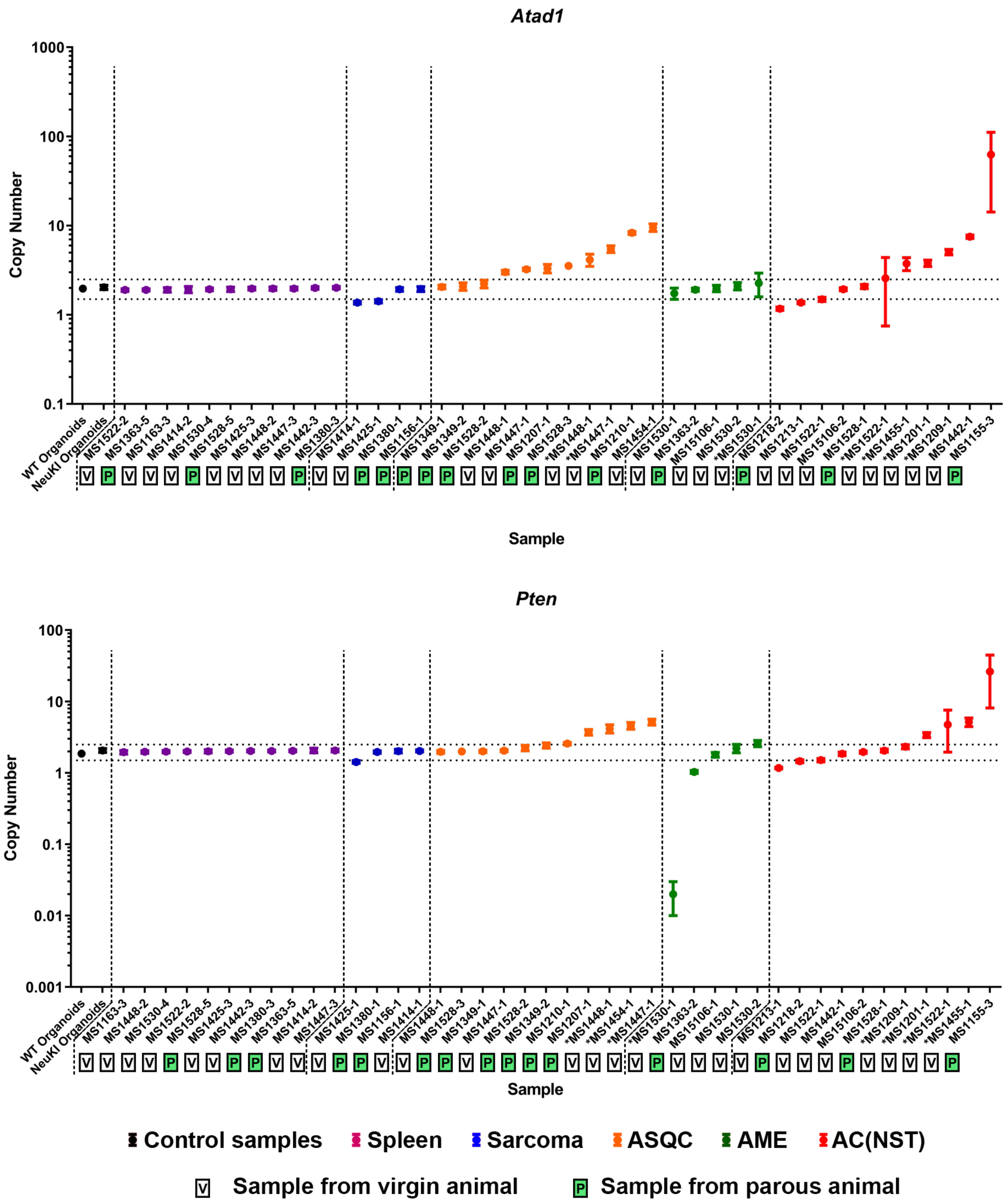
Atad1
1000
100
10
1
0.1
Copy Number
Sample
Pten
100
10
1
0.1
0.01
0.001
Copy Number
Sample
Control samples
Spleen
Sarcoma
ASQC
AME
AC(NST)
Sample from virgin animal
Sample from parous animal

Figure S4: Copy number changes in *Atad1* and *Pten* in *BlgCre-NeuKI* tumours. Copy number of *Atad1* (top) and *Pten* (bottom) (mean±95% confidence intervals of three technical repeats for each sample) in spleens and tumour samples from *BlgCre-NeuKI* mice and in wild type and *NeuKI* organoid controls (eight biological replicates each of three technical replicates) by ddPCR. Samples are arranged from lowest to highest amplification from left to right and colour coded to indicate sample type (Black, control; purple, spleen; blue, sarcoma; yellow, ASQC; green, AME; red, AC(NST)). *Indicates analysis carried out on DNA extracted from FFPE material. For some tumours, both snap frozen and FFPE material were tested. See **Table S10** for detailed results.

Disease Models & Mechanisms • Supplementary information

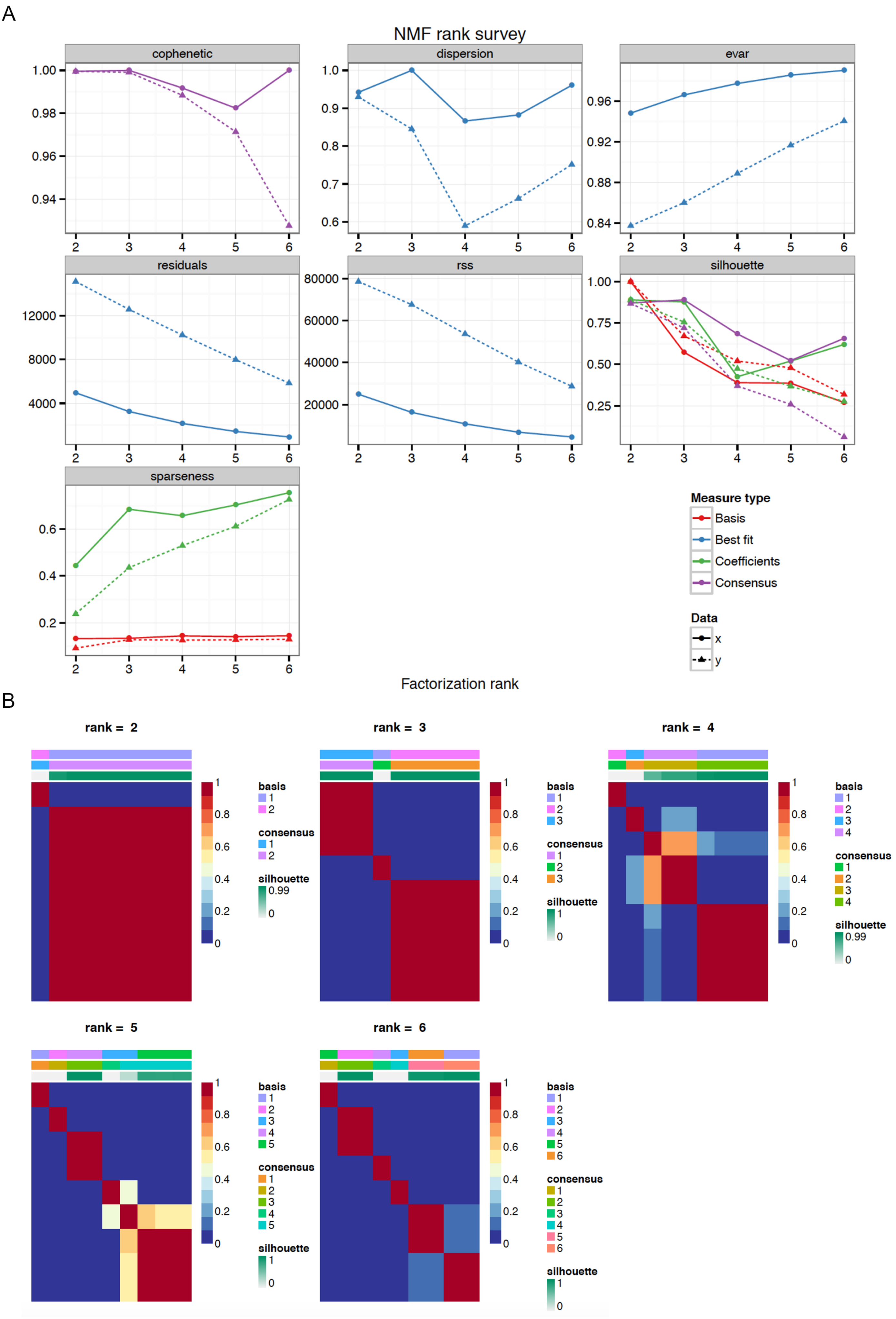
A
NMF rank survey
cophenetic
dispersion
evar
residuals
rss
silhouette
sparseness
Measure type
Basis
Best fit
Coefficients
Consensus
Data
x
y
Factorization rank
B
rank = 2
rank = 3
rank = 4
rank = 5
rank = 6
basis
consensus
silhouette

Figure S5: Non-negative Matrix Factorisation analysis of RNAseq data. Estimation of the rank by the R package *NMF* [18]. **(A)** Quality measures computed from 50 runs for each value of rank *k* across the expression dataset. **(B)** Consensus matrices ranked 2 – 6 computed from 50 runs for each value of rank *k* across the expression dataset. The top ranked matrix is shown in **Figure S6A**.

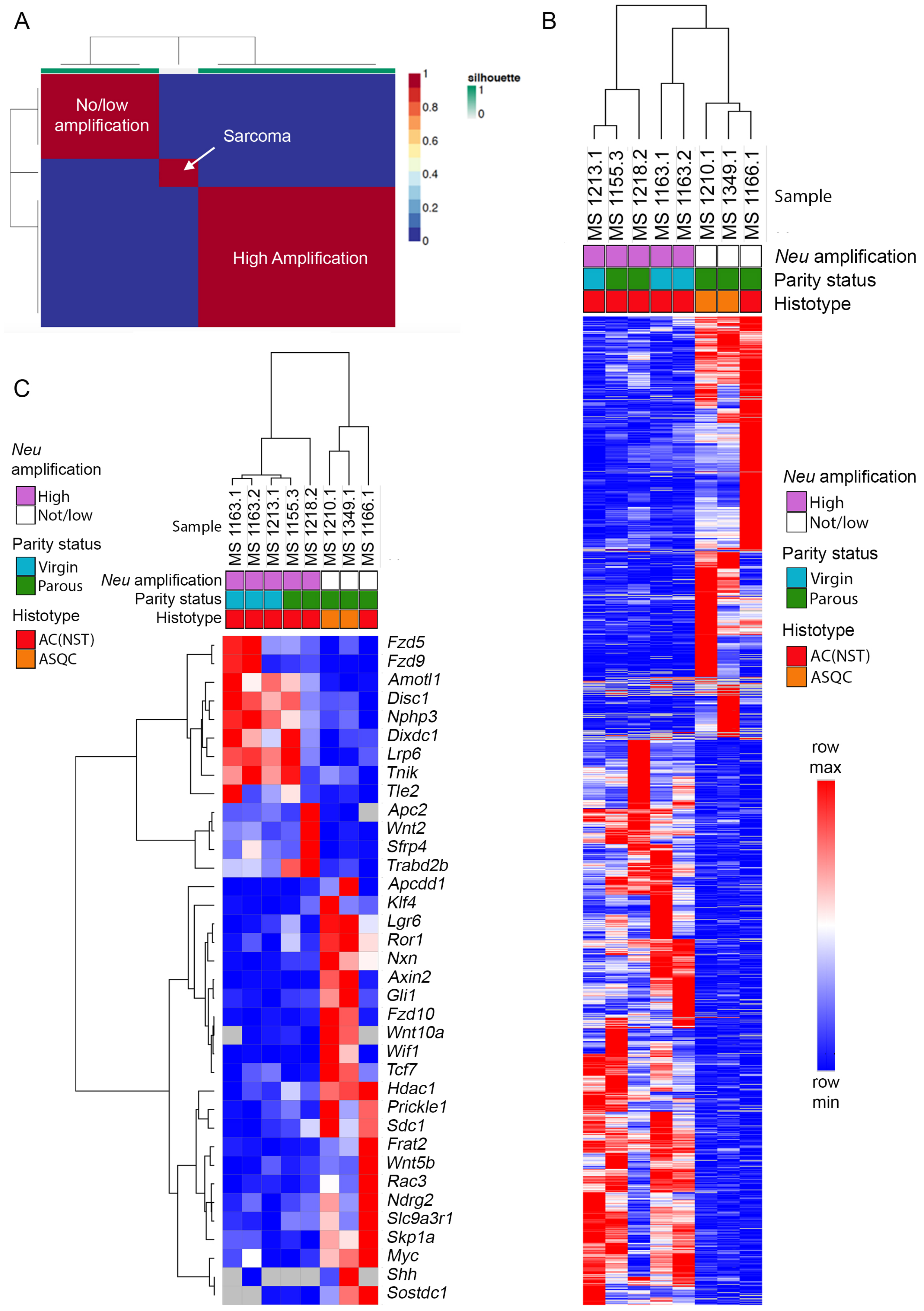

Disease Models & Mechanisms • Supplementary information

Figure S6: Unsupervised hierarchical clustering of RNAseq data. (A) Top ranked consensus matrix for cluster allocation analysis of RNASeq data using Non-negative Matrix Factorisation to identify correlations between tumour characteristics and gene expression. **(B)** Heatmap of results of unsupervised hierarchical clustering (use Broad Institute Morpheus tool with default parameters; clustering by both rows and columns) of normalized FPKM values for genes differentially expressed between not/low-amplified and high amplified samples. Neu amplification status (by qPCR), parity and histotype of each sample are indicated. **(C)** Heatmap of results of unsupervised hierarchical clustering of differentially expressed genes with the following GO (Bioprocess) / KEGG annotations: mmu04310:Wnt signalling pathway, GO:0016055~Wnt signalling pathway, GO:0060070~canonical Wnt signalling pathway, GO:0090090~negative regulation of canonical Wnt signalling pathway, GO:0090263~positive regulation of canonical Wnt signalling pathway. *Neu* amplification status (by qPCR), parity and histotype of each sample are indicated.

Disease Models & Mechanisms • Supplementary information

A)

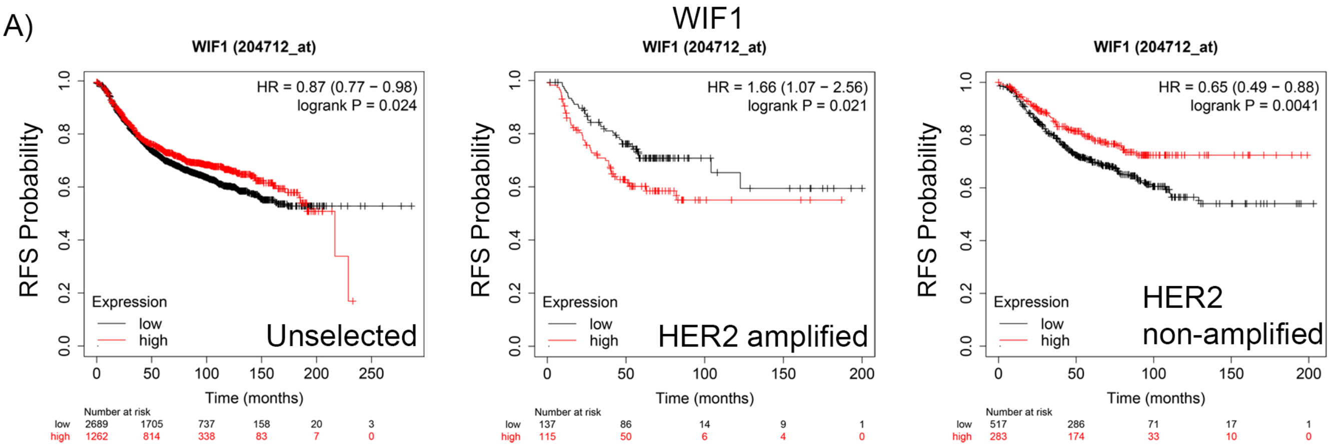

B)

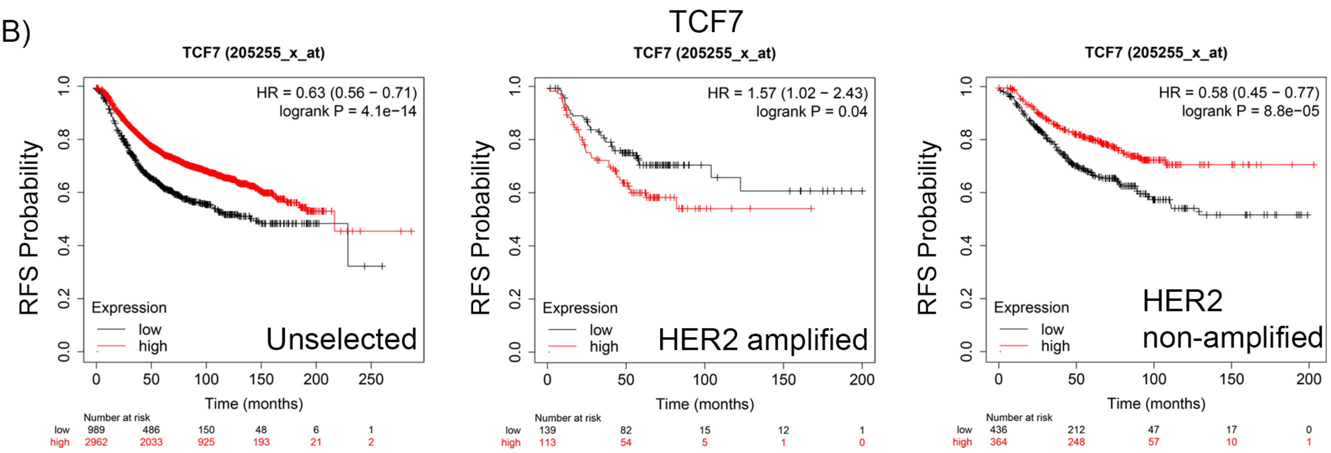

C)

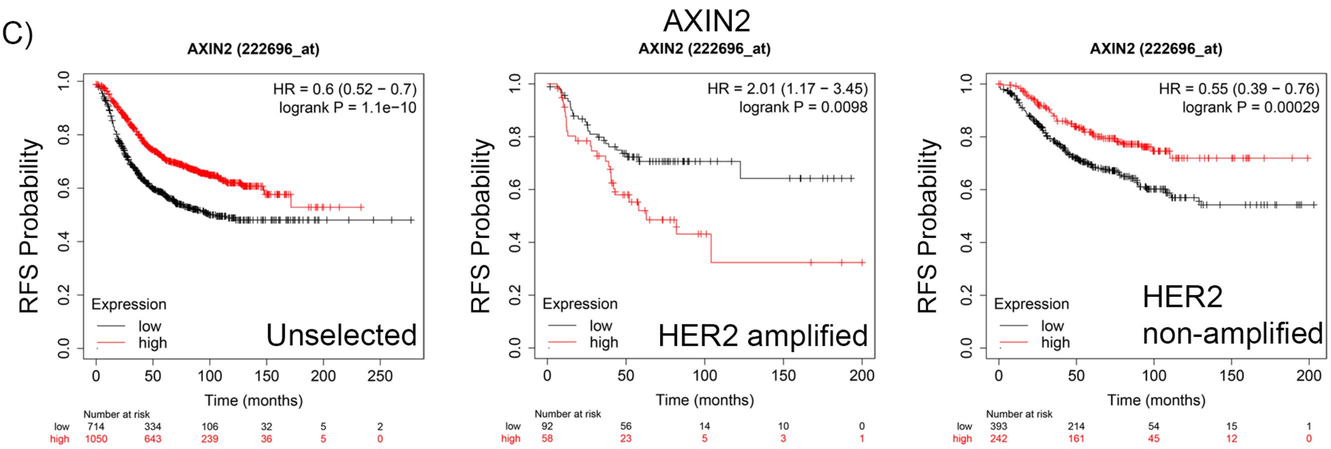

D)

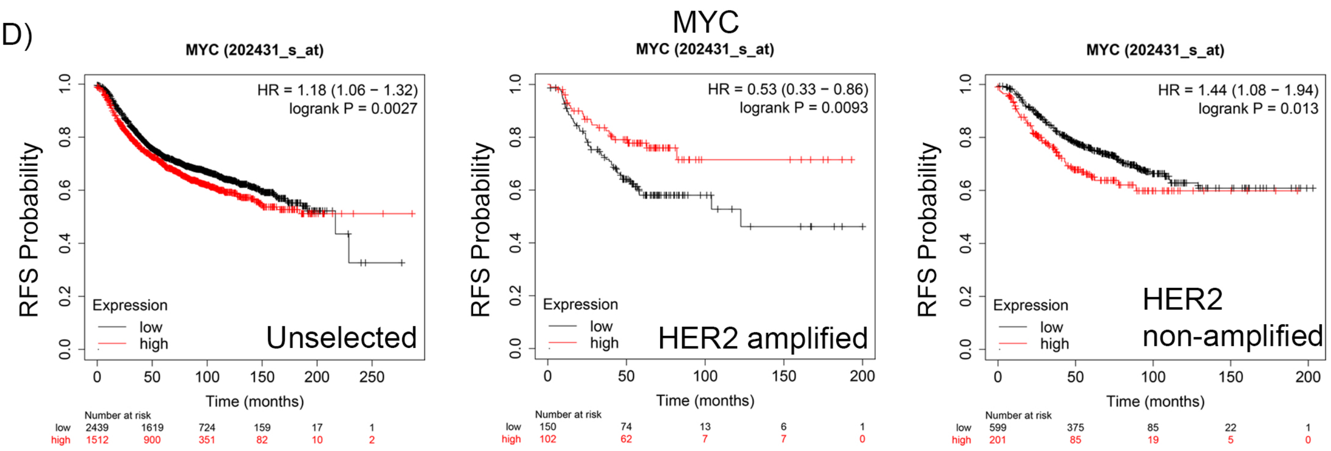

Disease Models & Mechanisms • Supplementary information

Figure S7: Relapse Free Survival (RFS) of HER2 amplified and non-amplified breast cancer stratified by canonical WNT target genes. Data from KM plotter for unselected breast cancers, HER2 amplified and HER2 non-amplified breast cancers stratified by expression of *WIF1* **(A)**, *TCF7* **(B)**, *AXIN2* **(C)** and *MYC* **(D)** using 'auto select best cut-off' mode.

Supplementary Tables

Table S1: Full details of tumour cohort animals included in the study. The database number of the animal, the block number of any sample taken for pathology, age, parity status, number of tumours, location and comments are provided. For a small number of cases histology blocks were missing.

Click here to download Table S1

Table S2: Probes for qRT-PCR, digital droplet PCR and genotyping.

Click here to download Table S2

Table S3: Full statistics matrix showing statistical differences between tumour cohorts. Statistics for survival curves (**Figure1A** and **1C**) are provided using both the Log Rank Mantel-Cox test and the Gehan-Breslow-Wilcoxon test. Statistics for differences between tumour numbers per animal **(Figure 1D)** used the Mann-Whitney test.

Click here to download Table S3

Table S4: Full descriptions of tumours which underwent detailed histological examination. Tumours were assessed as described previously [7,8]. All *K14Cre* and *BlgCre* tumours which were available underwent detailed examination. Only a selection of the *MMTV-NeuNT* tumours, chosen at random, underwent detailed examination. Scoring of staining for IHC markers (K14, K18, ERα, PR, p63) was by estimating percentages of positive cells within the tumour. Negative = no staining; 1 = <10% of cells positive; 2 = 10 – 50% cells positive; 3 = 50 – 75% cells positive; 4 = >75% cells positive.

Click here to download Table S4

Table S5: Details of samples used for exome sequencing and RNAseq. Sample numbers allocated by the sequencing facilities, tumour numbers, parity status, tumour phenotype and the amplification status of the locus by qPCR are provided, sorted by both tumour number and sequencing number for convenience. See also **Table S6** (detailed results of exome sequencing), **Table S7** (exome sequencing summary), **Table S8** (detailed results of CNV-by-exome analysis), **Table S9** (summary of CNV-by-exome analysis), **Table S11** (FPKM values for RNAseq), **Table S12** (differentially expressed genes) and **Table S13** (GO/KEGG analysis of differentially expressed genes).

Click here to download Table S5

Disease Models & Mechanisms • Supplementary information

Table S6: Detailed results of exome sequencing. Coding mutations identified are listed in the first sheet and the full Mutect and VEP outputs for each tumour are provided. Tumour IDs 11 to 32 refer to the IDs on Table S5 (B67-0011 to B67-0032). Summary data in **Table S7**.

Click here to download Table S6

Table S7: Summarised exome sequencing data.

Click here to download Table S7

Table S8: Detailed results of CNV-by-exome analysis. Summary data in **Table S9**. Results for the *Erbb2* locus are presented graphically in **Figure 3B**.

Click here to download Table S8

Table S9: Summarised CNV-by-exome analysis. Gains/losses in analysed tumours with an amplification log ratio >4.00 or a loss log ratio <-2.50. Genes in the predicted amplified/lost segment are indicated, together with a summary of their expression levels from the RNAseq data, in order to conclude whether or not the predicted genomic change has potential as a driver event.

Click here to download Table S9

Table S10: ddPCR analysis of *NeoR*, *Neu*, *Erbb2*, *Grb7*, *Mien1*, *Pten* and *Atad1*. Data for each locus are provided as a mean and 95% confidence intervals ('Error max' and 'Error min' based on triplicate technical replicates of each tumour and eight biological replicates, each of three technical replicates, for the wild type and *NeuKI* organoids) for the control organoid samples and the tumours. A summary sheet for the *NeoR* – *Neu* – *Erbb2* locus is also provided, and a sheet showing the concordance of results across different methods. The majority of analyses were carried out on snap frozen material, however for some samples only DNA extracted from FFPE sections was available. For four samples, the analysis was carried out on both. See **Figures S2 – S4** for graphical presentation of the data.

Click here to download Table S10

Table S11: Normalised FPKM values for tumours analysed by RNAseq. Differentially expressed genes are listed in **Table S12** and GO/Pathway analysis in **Table S13**.

Click here to download Table S11

Disease Models & Mechanisms • Supplementary information

Table S12: Significantly differentially expressed genes in high amplified and non/low amplified tumours. Relative expression levels for all genes are shown as well as genes with a significant (FDR P value <0.05 and a fold change >2) expression difference comparing tumours with a highly amplified with a non/low amplified *Neu* allele. Genes significantly upregulated in highly amplified tumour are, by definition, significantly downregulated in non/low amplified tumours, and vice versa.

Click here to download Table S12

Table S13: Functional annotation and functional annotation clustering of genes differentially expressed in highly amplified and non/low amplified tumours. Clustering by DAVID v6.8 using GO Bioprocess and KEGG pathway annotation. Annotations of particular interest are highlighted in red. See also **Table 1**.

Click here to download Table S13

Table S14: Genomic locations of differentially expressed genes. Genomic locations by chromosome number and locus start site from Mouse Genome Informatics database (JAX).

Click here to download Table S14

Table S15: qrtPCR analysis results for β-catenin expression and canonical WNT target genes. See **Figure 5** for graphical presentation of the data.

Click here to download Table S15

Disease Models & Mechanisms • Supplementary information
